# Animal medical systems from *Apis* to apes: history, recent advances and future perspectives

**DOI:** 10.1111/brv.70060

**Published:** 2025-07-27

**Authors:** Michelina Pusceddu, Michael A. Huffman, Stephane Knoll, Ana Helena Dias Francesconi, Ignazio Floris, Alberto Satta

**Affiliations:** ^1^ Department of Agricultural Sciences University of Sassari Viale Italia 39a Sassari 07100 Italy; ^2^ National Biodiversity Future Center (NBFC) Piazza Marina 61 Palermo 90133 Italy; ^3^ Institute of Tropical Medicine Nagasaki University 1‐12‐4 Sakamoto Nagasaki 852‐8523 Japan; ^4^ Department of Anthropology University of Sri Jayewardenepura Nugegoda 10100 Sri Lanka

**Keywords:** medical behaviours, medication behaviours, life history, sociality, ecological adaptations

## Abstract

Animal medical systems encompass a wide range of behaviours aimed at maintaining or improving health. It has become clear that these behaviours are not limited to animals treating themselves (self‐medication) but also include the treatment of group members, resulting in the adoption of the more inclusive term “animal medication”. Behaviour with the intent to avoid, reduce the impact, or otherwise treat disease transmission, rather than the use of medicinal substances, can be described as *medical behaviours*. However, most behaviours described here involve the ingestion or application of items with medicinal properties to oneself or the application of items to others or their temporary or permanent communal living spaces like nests or burrows, hereto named *medicinal or medication behaviours*. This review begins with a historical overview of the field, showcasing an increasing awareness of the wide diversity of taxa exhibiting animal medication and elucidating the development of criteria used to define and categorise such behaviours across the animal kingdom. A thorough synthesis of recent research is presented, by providing critical reflection that challenges conventional notions and emphasises the significance of sociality and ecological context. To this end, medical systems are explored by using numerous examples, thus highlighting the diverse strategies animals employ to maintain health and improve fitness, ranging from honey bees foraging on antimicrobial resin to control hive disease to apes ingesting small amounts of toxic secondary compounds to control parasite infection. The understanding of how animals maintain their health through medical strategies offers valuable insights into the evolutionary origin and complexity of the drivers behind these behaviours. Evidence suggests that advanced cognition is not necessarily a prerequisite because innate mechanisms are likely involved in the expression of these behaviours across the animal kingdom. By highlighting the importance of life‐history traits and ecological context in predicting animal medical systems, we reassess the presumed primary drivers of these adaptations. Finally, this review raises important questions about animal medical systems, including the universality of the mechanisms involved, the evolutionary significance of parasite pressure, and the ecological implications of this suite of behaviours. By addressing these complexities, this review provides a nuanced understanding of animal medical systems and highlights avenues for future research in this field.

## I. INTRODUCTION

In nature, the control or avoidance of disease constitutes a crucial fitness strategy, necessitated by persistent challenges posed by pathogens and parasites to all animal species. Five behavioural lines of defence employed by animals were first described by Hart (1990, 2011) in response to pathogens and parasites, drawing parallels to fundamental principles of the human medical system: quarantine, vaccination, nursing care, and the use of medicinal substances. These strategies were summarised as follows. (*i*) *Avoiding and removing pathogens and parasites*: avoiding contact with infective agents on substrates or contaminated food/water sources, grooming to remove ectoparasites, fly‐repelling behaviours such as scratching with their hind legs or using swatting stick tools or other plant material to repel biting insects. Notably, animals apply saliva as a natural antiseptic for cleaning wounds, their genitals after copulation to protect against sexually transmitted infections, or nipples before nursing to protect newborns with underdeveloped immune systems. (*ii*) *Quarantine*: repelling strangers or peripheralizing sick group members from the colony or social group who may be carrying pathogens. This behaviour is often observed as territorial defence and, sometimes, as cannibalism of sick offspring to protect the rest of the litter. (*iii*) *Immunisation*: acquiring natural immunity through gradual exposure to pathogens. Behaviours that facilitate this process include controlled interactions with strangers, young animal exposure to potential diseases, and immune potentiation through immune‐sensitising doses of pathogens by social interactions or from the environment. (*iv*) *Caring of the sick and injured*: supporting ill or injured group members. These behaviours include food provisioning, physical assistance, care for a sick individual's dependent offspring, and providing companionship. (*v*) *Use of medicinal herbs*: using plants prophylactically to prevent or therapeutically to treat existing conditions. For this, animals have evolved the ability to use a diverse array of bioactive substances, derived from plants or other animals, fungi, and inorganic materials such as soil (de Roode & Huffman, 2024).

The possibility that some animals may benefit from the ingestion of toxic plant secondary compounds for their medicinal value was first raised by Janzen (1978). Primatologists paid attention to this work and cited it when they began to present evidence for the non‐nutritional swallowing of whole leaves (Wrangham & Nishida, 1983), the prophylactic ingestion of edible berries of *Balanites aegyptiaca*, with known antiparasitic properties by Hamadryas baboons (*Papio hamadryas*) and olive baboon (*P. anubis*) × Hamadryas baboon hybrids living in areas with high risk of infection with bilharzia (*Schistosoma mansoni*) (Phillips‐Conroy, 1986), and the therapeutic use of *Vernonia amygdalina*, a well‐known human antiparasitic medicinal plant, by chimpanzees (*Pan troglodytes*) suffering from parasite infection or related symptoms (Huffman & Seifu, 1989; Huffman *et al*., 1993). The ingestion of *V. amygdalina* by chimpanzees is considered the earliest and most convincing evidence for the mitigation of the impact of parasites and pathogens (Huffman, 1997) and became the focus of early theoretical discussions (Clayton & Wolfe, 1993; Glander, 1994; Huffman, 1997; Lozano, 1998). In the early 1980s, animal self‐medication encountered some scepticism due to the circumstantial or anecdotal evidence presented early on (Sapolsky, 1994; Lozano, 1998; Engel, 2002), but self‐medication is now widely recognised to occur across the animal kingdom (Huffman, 2022).

Initially, the examples of self‐medication behaviour, mostly limited to primates, were presented by the popular press and parts of the scientific community as evidence of behaviours uniquely human‐like and therefore requiring advanced cognitive abilities. This was perhaps encouraged by the early popularisation of the field with the term “zoopharmacognosy”, based on the Latin scientific terminology *zoo* (animal), *pharmaco* (medicine), and *gnosy* (knowing) (Rodriguez & Wrangham, 1993). This perception gradually changed as more solid evidence accumulated from other taxa, such as birds (Clayton *et al*., 2010; Morozov, 2015; Bush & Clayton, 2018), reptiles and amphibians (McCracken & Forstner, 2006; Villanueva *et al*., 2022; Williams *et al*., 2022), and insects (e.g. reviewed by de Roode, Lefèvre & Hunter, 2013; Abbott, 2014; Erler *et al*., 2024). The discovery of the role of innate responses in the medicative behaviour of animals with supposedly less‐advanced cognitive function, such as insects, challenged the conventional wisdom of the day (Singer, Mace & Bernays, 2009; de Roode *et al*., 2013; de Roode & Hunter, 2019).

As new behaviours and species exhibiting them have been discovered and described, our understanding of self‐medication has rapidly expanded. Classically, the two major modes of medicative behaviour were *therapeutic medication* (treating existing infections) and *prophylactic medication* (preventing infections) (de Roode & Huffman, 2024; Erler *et al*., 2024), whereby medicinal substances can be ingested, applied to the body (*anointing*) or placed in an animal's living area (*fumigation*). Later findings expanded these criteria to encompass behaviours resulting from an increased infection risk called *passive prevention*, a more nuanced form of prophylactic self‐medication regarding the impact of parasite infection on seasonal medicinal food selection patterns (e.g. Huffman 1997, 2016; MacIntosh & Huffman, 2010; Viviano *et al*., 2022). However, the classification of behaviour as being either therapeutic or prophylactic is not always straightforward, as some behaviours can serve both purposes simultaneously (Huffman, 1997; de Roode & Hunter, 2019). In addition, it has become necessary to define the recipients of care in more detail, due to the complexity of animal medical systems being revealed. These categories are *self‐medication* (medicating oneself), *allo‐medication* (medicating other individuals, when kin relationships are not known), *kin medication* (medication of genetic relatives when kin are known), and *social medication* (medicating the entire group or colony resulting in benefits for all members) (de Roode & Huffman, 2024; Erler *et al*., 2024). Some of the newest examples of allo‐medication occur in a wide range of taxa, from insects (de Roode & Hunter, 2019), to birds (Suárez‐Rodríguez & Garcia, 2017), to mammals (Mascaro *et al*., 2022).

Foundational criteria of self‐medication are outlined as (*i*) the intentional use of medicinal substances by the host (or medicator), (*ii*) the detrimental effect of such substances on parasites (broadly defined to include viruses, bacteria, protozoans, helminths, arthropods, and fungi), and (*iii*) subsequent fitness benefits for the host (Huffman & Seifu, 1989; Clayton & Wolfe, 1993; Huffman *et al*., 1993; Huffman, 1997; Forbey *et al*., 2009). Research on insects has expanded these criteria, suggesting similarities and some interesting possible differences between insects and other animals. Seminal works by Singer *et al*. (2009), de Roode *et al*. (2013), and de Roode & Hunter (2019) have been pivotal for the recognition of insect self‐medication (Abbott, 2014). Singer *et al*. (2009) expanded on these criteria by introducing the concept of adaptive plasticity, emphasising that, while self‐medicating behaviour must improve the fitness of infected animals, the same behaviour comes at a cost for uninfected individuals. This is an important concept as it distinguishes some forms of self‐medication from flexible dietary choices without any cost, thus setting a higher standard for defining self‐medication in insects (Abbott, 2014). However, this does not necessarily apply to all kinds of self‐medicative behaviour, such as passive prevention in vertebrate species.

de Roode *et al*. (2013) presented a nuanced framework for insects that includes the relevance of the behaviour to the natural environment of the host and emphasises that a self‐medicative behaviour does not necessarily need to reduce parasite infection or growth; instead, insect hosts may use medication to increase tolerance of infections. These authors contributed significantly to the field by arguing that self‐medicating behaviour should not be limited to those benefiting the host itself but can also apply to its genetic kin or other relatives within the social context of that species (*transgenerational and social medication*), creating an inclusive fitness framework for self‐medication for species that do not perform direct parental care (de Roode *et al*., 2013). This emphasis on the medication of others has resulted in the adoption of a more general term “animal medication” as opposed to just “animal self‐medication” (Erler *et al*., 2024; de Roode & Huffman, 2024). Individuals of a given species can perform both medication types.

**Fig. 1:**
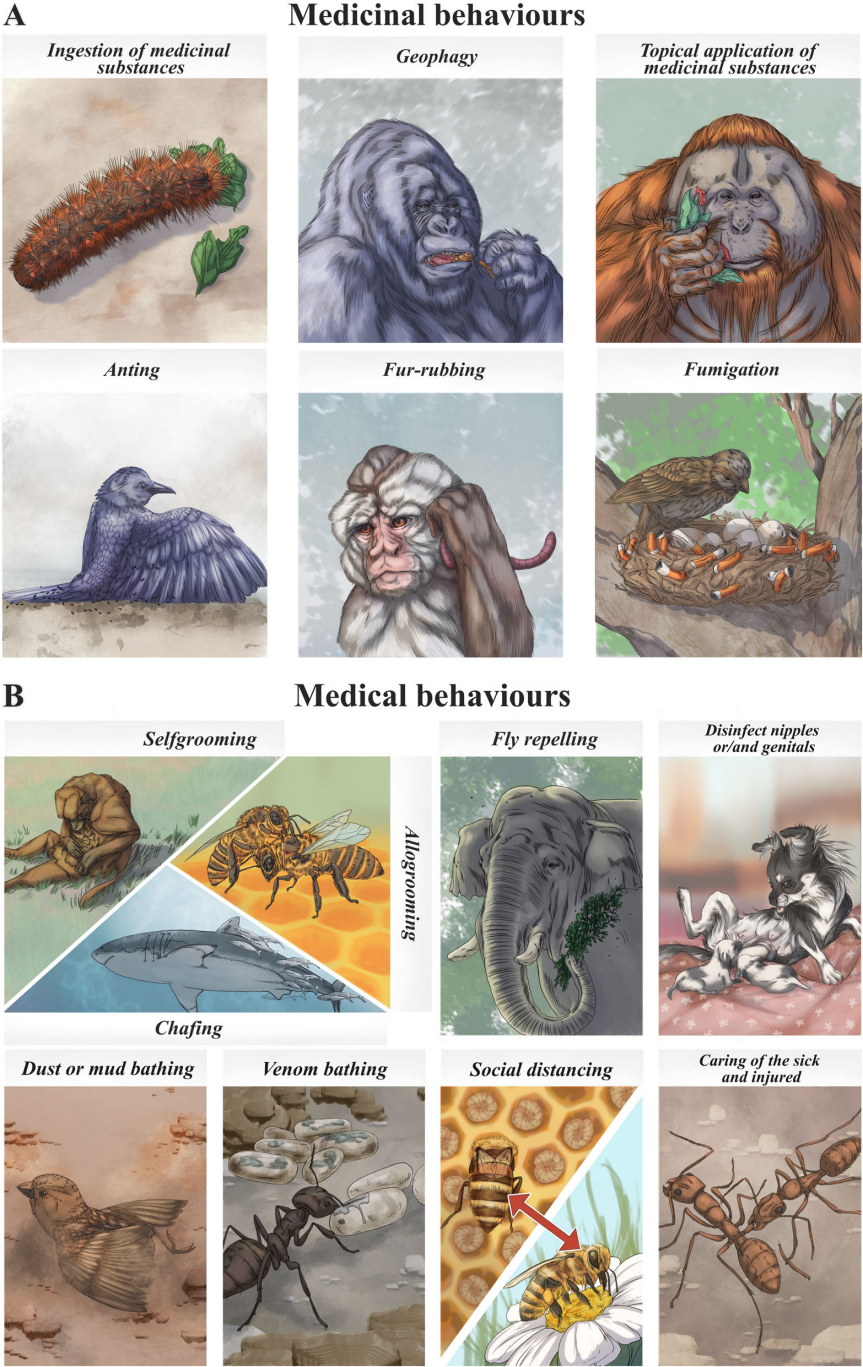
Animal medical systems display a wide range of behaviours aimed at maintaining or improving health. Medication or medicinal behaviours (A) involve the ingestion or application of substances (obtained from plants, fungi and other animals) with medicinal properties to oneself, to others, or to their living environment. Medical behaviours (B) are those intended to prevent, reduce the impact, or otherwise treat disease transmission, rather than those involving the use of medicinal substances.

In this comprehensive review, we illuminate instances both of *medication or medicinal behaviours* (the use of medicinal substances – plant, mineral, or animal – to treat illness or injury; Fig. 1A) and *medical behaviours* (acts other than the application of medicinal substances to the body with the intent to avoid, reduce the impact, or otherwise treat disease transmission from infective agents or sick individuals; Fig. 1B), amidst animal life diversity, emphasising the parallels among widely different taxa. By using this comparative lens and recognising that current studies are often confined to single models, this review aspires to an inclusive approach, by exemplifying not only the diversity across species, but also the shared threads that weave through the varied social and individual models of medical systems. By emphasising the adaptability of these behaviours and their relevance to diverse ecological settings, we aim to provide a comprehensive understanding of animal medical systems based on the most relevant data. Ultimately, we raise challenging questions to stimulate critical reflection and push the existing boundaries of the field further forward.

## II. VERTEBRATES

### (1) Mammals and birds

Much of the evidence of self‐medication originally stemmed from the Eastern chimpanzees (*Pan troglodytes schweinfurthii*), bonobos (*P. paniscus*), and eastern lowland gorillas (*Gorilla beringei graueri*) (Glander, 1994; Huffman & Wrangham, 1994; Huffman, 1997, 2003). Notably, the identification of self‐medication in these animals early on was facilitated by sympatry (sharing of ecological niches) and the striking parallels in behaviours between humans and other primate species (e.g. utilisation of similar remedies for comparable illnesses). This may have led some researchers to assume that advanced cognitive abilities were needed to perform self‐medication.

Two widely acknowledged examples of therapeutic animal self‐medication arose from observations of chimpanzees at Gombe National Park and Mahale Mountains National Park in western Tanzania along the shore of Lake Tanganyika. One is called leaf‐swallowing behaviour and consists of the folding and swallowing, without chewing, of rough, hispid leaves that later are defecated as an undigested mass of leaves. This unusual feeding behaviour was first described by Wrangham & Nishida (1983), although its therapeutic value was not recognised. Rodriguez *et al*. (1985) proposed that a pharmacological antiparasitic property could explain the consumption of *Aspillia* spp., but critical evaluation from the field and laboratory provided no supporting evidence (Huffman *et al*., 1996; Page *et al*., 1997). Later, leaf‐swallowing behaviour was linked to the physical expulsion of adult nematodes and cestode proglottids (Wrangham, 1995; Huffman *et al*., 1996; Huffman, 1997). Based on field and laboratory evidence, a mechanism was proposed for the antiparasitic behaviour (Huffman *et al*., 1996; Page *et al*.,1997; Huffman & Caton, 2001). Leaf‐swallowing is now recognised to occur in many primate species, including all sub‐species of chimpanzees, bonobos, the eastern lowland gorillas and western lowland gorillas (*Gorilla gorilla gorilla*), and lar gibbons (*Hylobates lar*) (Barelli & Huffman, 2017) as well as other vertebrates, including brown bears (*Ursus arctos*), snow geese (*Anser caerulescens*), and Chinese lesser civets (*Viverricula indica*) (Huffman, 1997; Su, Su & Huffman, 2013).

The second early example of therapeutic self‐medication in a non‐human species is bitter pith chewing, involving the deliberate ingestion of highly bitter juices from the pith of *V. amygdalina* by chimpanzees parasitised by the nodule worm *Oesophagostomum stephanostomum*, to combat infection and its associated symptoms (Huffman & Seifu, 1989; Huffman *et al*., 1993; Huffman, 1997). Extensive field and laboratory investigations into bitter pith chewing resulted in the formulation of a standardised methodology for empirically establishing therapeutic self‐medication: (*i*) identifying the treated disease or symptom(s); (*ii*) distinguishing therapeutic agents from regular food items; (*iii*) documenting improved health following self‐medication; and (*iv*) providing evidence of pharmacological activity of the ingested compounds relevant to ameliorated disease or symptoms (Huffman, 1997, 2022). Investigating self‐medication in primates has various practical and ethical limitations. Indeed, systematically observing self‐medicating behaviours in free‐ranging primates is difficult because sick individuals are often difficult to find, due to the fact that they tend to move away from the group. Furthermore, *in vivo* trials with primates may be neither practical nor ethical (Huffman, 1997; Lozano, 1998; Hutchings *et al*., 2003).

Research on domesticated herbivores is ethically and logistically easier, as observational studies and manipulative experiments are much more feasible (Villalba & Provenza, 2007). Furthermore, these studies are of considerable economic interest because parasite infections, particularly by gastrointestinal nematodes, are productivity‐impacting illnesses in farm animals (Villalba *et al*., 2014). Various experiments have demonstrated apparent self‐medicating behaviour in goats (*Capra hircus*) and sheep (*Ovis aries*) (see review by Villalba *et al*., 2014; Torres‐Fajardo *et al*., 2019), where parasitised animals seemingly ingest plants for their secondary compounds. For instance, kid goats (*Capra aegagrus* f. *hircus* “Mamber”) inoculated with gastrointestinal nematodes significantly increased their intake of *Pistacia lentiscus* (Landau *et al*., 2010; Amit *et al*., 2013), a shrub with antiparasitic properties (Azaizeh *et al*., 2012), indicating therapeutic self‐medication. Interestingly, this dietary selection behaviour seems to be learned from observing the behaviour of the mother or others in the group, demonstrating social learning as part of self‐medication in these animals (Glasser *et al*., 2009), as also shown for leaf‐swallowing in chimpanzees (Huffman & Hirata, 2004; Huffman *et al*., 2010). Experimental evidence has also demonstrated self‐medication through individual learning in domestic sheep, as animals learned to consume chemicals mitigating experimentally administered dietary toxins (Villalba, Provenza & Shaw, 2006).

The ingestion of medicinal substances to counteract dietary toxins has been observed across multiple taxa. Zanzibar red colobus monkeys (*Piliocolobus kirkii*) will actively consume burned wood (charcoal), which binds to secondary compounds and reduces their toxicity. This enabled the colobus to gain maximum nutritional benefits from the plants available, allowing incorporation into their diet of non‐native plants at times of low availability of native plants (Cooney & Struhsaker, 1997). In human medicine, oral administration of activated charcoal serves as a first‐line intervention to prevent the gastrointestinal absorption of toxic substances, and is used in cases of poisoning and drug overdoses (Cooney & Struhsaker, 1997). While charcoal consumption is relatively uncommon, geophagy, the ingestion of clay or earth, has the same function and is widespread among primates (Krishnamani & Mahaney, 2000; Pebsworth *et al*., 2019; Borruso *et al*., 2021) and many other animal taxa (Villalba & Provenza, 2007; Costa‐Neto, 2012; Downs, Bredin & Wragg, 2019). Studies suggest that mountain gorillas (*Gorilla beringei beringei*) may ingest clay, which has water‐holding properties, as a remedy against diarrhoea (Mahaney, Watts & Hancock, 1990; Mahaney, Hancock & Aufreiter, 1995), as is also practised by humans (Ferrell, 2008).

In addition to ingesting medicinal substances, vertebrates apply them externally (fur rubbing, anting, and fumigation), by utilising compounds from plant, animal, and inorganic sources. For example, fur rubbing is a form of anointing behaviour, described in Central and South American primates like capuchin monkeys (*Cebus capucinus*), black lemurs and red‐fronted lemurs (e.g. *Eulemur macaco, Eulemur rufifrons*), white‐nosed coatis (*Nasua narica*), and brown bears (Gompper & Hoylman, 1993; Baker, 1996; Birkinshaw, 1999; DeJoseph *et al*., 2002; Lynch Alfaro *et al*., 2012; Peckre *et al*., 2018; Blaise *et al*., 2023), where material is applied directly to the fur or skin purportedly for antiparasitic, insect‐repelling, antifungal, antibacterial, and/or anti‐inflammatory properties (Baker, 1996; Huffman, 1997; Lozano, 1998; DeJoseph *et al*., 2002; Verderane *et al*., 2007; Morrogh‐Bernard *et al*., 2017). Orangutans (*Pongo pygmaeus*) apply a galenic preparation (also known as defensive mixology) of saliva and *Dracaena cantleyi* leaf extracts to various body parts, presumably for the treatment of joint and muscle inflammation and pain, similar to local Indigenous Peoples' practices (Morrogh‐Bernard, 2008; Morrogh‐Bernard *et al*., 2017). A male Sumatran orangutan was observed self‐treating a facial wound by using a plant with biologically active properties (Laumer *et al*., 2024). Thus there is potential for external use of these bioactive substances as a therapeutic dressing for bacterial infections or to enhance wound healing. Capuchin monkeys rub scented plant materials (e.g., citrus) throughout their fur apparently to repel ectoparasites and biting insects (Baker, 1996, 2017; DeJoseph *et al*., 2002). White‐nosed coatis rub themselves with resin scraped from the bark of *Trattinnickia aspera* trees to combat fleas, ticks, and lice, and to repel mosquitoes (Gompper & Holyman, 1993). Besides anointing themselves, animals also engage in social or allo‐medication by topical application to the fur of conspecifics (Huffman, 1997; Bowler *et al*., 2015; Morozov, 2015). This may facilitate application of substances to parts of the body that individuals are unable to reach by themselves.

A notable variant of fur rubbing is observed in various capuchin species and lemurs that rub gently bitten millipedes together with saliva through their fur, presumably to elicit their defensive secretions that are then used as a repellent against hematophagous arthropods (Valderrama *et al*., 2000; Weldon *et al*., 2003; Peckre *et al*., 2018; Medeiros *et al*., 2020). Similarly, capuchin monkeys rub carpenter ants (*Camponotus* spp.) into their fur, presumably to control ectoparasites (Longino, 1984; Verderane *et al*., 2007). An alternative non‐medicinal social interaction hypothesis has been proposed for group fur rubbing in captive tufted capuchins (*Sapajus apella*), where this behaviour appeared to be significantly affected by kinship and dominance (Leca, Gunst & Petit, 2007). However, responses to chemical stimuli in plant or insect material that induce medicative behaviours, such as fur‐rubbing behaviour and leaf‐swallowing, in captivity in many species may simply represent “out‐of‐context” expression of adaptive predispositions to perform such behaviours in both invertebrates and vertebrates (Huffman & Hirata, 2003, 2004; Huffman *et al*., 2010; Uenoyama *et al*., 2021).

Anting has been recorded in over 200 bird species, where individuals either actively rub formic acid‐producing ants (such as carpenter ants) through their plumage or passively allow the ants to crawl through it (Simmons, 1957; Morozov, 2015). The ants' acid is thought to help maintain the bird's plumage and/or act as an antiparasitic agent against mites and lice (Clayton & Wolfe, 1993; Morozov, 2015). Birds also use a variety of arthropod species, including millipedes, and aromatic plant material, such as citrus, to perform this behaviour (Morozov, 2015).

Dust‐bathing (or dusting) is another self‐grooming behaviour of birds with antiparasitic effects. Dust‐bathing involves bathing in inorganic material, such as sand or loose soil, which adheres to their feathers and skin, thus suffocating and removing lice (Clayton *et al*., 2010; Bush & Clayton, 2018). Bearded vultures (*Gypaetus barbatus*) apply iron‐oxide‐rich soil to their plumage presumably as a preventive measure against harmful bacteria on the carcasses they consume (Arlettaz *et al*., 2002; Tributsch, 2016), although this hypothesis remains controversial (Margalida, Almirall & Negro, 2023). Mammals also practice dust‐bathing (Coppedge & Shaw, 2000; Rees, 2002). Ungulates, such as elephants (*Elephas maximus*), rhinoceros (*Rhinoceros unicornis*), buffaloes (*Bubalus bubalis*), bison (*Bos bison*), and wild boar (*Sus scrofa*), frequently engage in mud‐bathing for many of the same reasons as birds: thermoregulation, protection from biting insects and/or the removal of parasites (McMillan, Cottam & Kaufman, 2000; Joshi, 2009; Bracke, 2011).

Animals can also apply medicinal substances to their immediate environment, a phenomenon termed fumigation (Hemmes, Alvarado & Hart, 2002; Huffman, 2019). A notable example of this behaviour includes incorporation of fresh, green vegetation into the nests or dens by birds and mammals including greater short‐nosed fruit bats (*Cynopterus sphinx*) and some great ape species (Wimberger, 1984; Rajasekar, Chattopadhyay & Sripathi, 2006; Bush & Clayton, 2018; De la Fuente *et al*., 2022). These animals select plant material for its aromatic secondary compounds, which can repel or even eliminate parasites (Wimberger, 1984; Dumbacher & Pruett‐Jones, 1996; Weldon & Carroll, 2006; Scott‐Baumann & Morgan, 2015; Bush & Clayton, 2018). For instance, European starlings (*Sturnus vulgaris*) line their nests with wild carrot (*Dauscus carota*) or fleabane (*Erigeron philadelphicus*), known for their mite‐suppressing and repelling properties (Clark & Mason, 1985, 1988; Clark, 1991; Panagiotakopulu *et al*., 1995). Insertion of pine greenery into Bonelli's eagle (*Hieraaetus fasciatus*) nests reduces the number of blowfly larvae and increases host reproductive success (Ontiveros, Caro & Pleguezuelos, 2008). Because the addition of green vegetation to nests starts before chicks hatch (Wimberger, 1984), this behaviour should be considered as therapeutic self‐medication for parents and allo‐medication for their offspring to ward off ectoparasites infesting the nest. An intriguing variation of this behaviour involves birds using nicotine‐infused cigarette butts, with demonstrated antiparasitic properties, to line their nests. House sparrows (*Passer domesticus*) and house finches (*Carpodacus mexicanus*) collect and incorporate discarded cigarette butts into their nests (Suárez‐Rodríguez, López‐Rull & Garcia, 2013; Suárez‐Rodríguez & Garcia, 2017). Nicotine is a widely used insecticide (Rodgman & Perfetti, 2013), and was shown to offer protection against mites and, potentially, other ectoparasites (Suárez‐Rodríguez & Garcia, 2014, 2017). The seeking out and use of cigarette butts suggests an adaptation to the urban environment, possibly in response to the absence of natural materials the species would normally utilise in a non‐urban setting, or as an adaptation to a more efficient material for parasite control (Reynolds *et al*., 2019).

### (2) Fishes, amphibians and reptiles

Currently, information on medical systems in reptiles and amphibians is scarce (Pandey & Verma, 2017; Sauer *et al*., 2019). It is well known that certain frog species (e.g. Dendrobatidae) sequester alkaloid defences from their arthropod diet and secrete them through cutaneous glands as a defence against predators (Saporito *et al*., 2007, 2009, 2012). These sequestered toxins also possess antibacterial (Hovey *et al*., 2018), potential antiparasitic (Weldon *et al*., 2006; Grant *et al*., 2012), and antifungal (Macfoy *et al*., 2005; Mina *et al*., 2015) properties. We consider this to be a form of prophylactic self‐medication. Additionally, some amphibians provide their offspring with alkaloid defences originating from their diet (Hayes *et al*., 2009; Gall *et al*., 2011; Villanueva *et al*., 2022). Frogs of the genus *Oophaga* provide their developing tadpoles with trophic, unfertilised eggs containing sequestered toxins, thus nourishing and offering protection to their offspring (Stynoski *et al*., 2014b; Fischer *et al*., 2019; Saporito *et al*., 2019; Brooks, James & Saporito, 2022; Villanueva *et al*., 2022). If these toxins confer protection to tadpoles against parasites comparable to their effectiveness against predators (Stynoski, Shelton & Stynoski, 2014a; Stynoski *et al*., 2014b), this could be considered an example of kin medication. However, their antimicrobial activity has yet to be demonstrated.

Similarly, research on tetrodotoxin in newts (*Taricha granulosa* and *T. torosa*; Johnson *et al*., 2018) and puffer fish (*Lagocephalus sceleratus*; Alabssawy, 2017) revealed an apparent protective function of this substance against micro‐ and macroparasites, in addition to its widely acknowledged role against predators. However, the precise origin of tetrodotoxins in these animals, whether endogenous, exogenous, or both, remains uncertain (Chau, Kalaitzis & Neilan, 2011; Itoi *et al*., 2018; Vaelli *et al*., 2020; Gall *et al*., 2022). The use of sequestered toxins for defensive purposes has also been documented in toads (*Melanophryniscus klappenbachi*) and snakes (*Rhabdophis tigrinus*) (Hutchinson *et al*., 2007; Savitzky *et al*., 2012; Mebs, Pogoda & Toennes, 2018), although their potential antiparasitic function remains largely unexplored (Rodríguez *et al*., 2017). Many amphibians (e.g. *Speleomantes* spp.) possess defensive cutaneous secretions with antiparasitic properties (Rollins‐Smith, 2009; Pasmans *et al*., 2013; Smith *et al*., 2018). Nevertheless, the origin and nature of the defensive compounds in these secretions is debated or, at least, appears to vary among species. Some studies propose exogenous bacterial metabolites to be the protective agent (Brucker *et al*., 2008a, 2008b), while others point to endogenous peptides (Smith *et al*., 2018). Whether the use of endogenous bioactive substances should be counted as medicative behaviour is still under debate, but we include it herein as as part of the animal medical system as it appears to perform a similar function as exogenous substances.

Geophagy also occurs in reptiles, amphibians, and fishes (Jain *et al*., 2008; Mamillapalli, Jujjavarapu & Kantamneni, 2016; Terebiznik *et al*., 2020). For example, individuals of *Bufo margaritifer* intentionally ingest mud, which may be a means to counteract toxins present in their predominantly ant‐based diet (McCracken & Forstner, 2006). However, in these vertebrates, geophagy is also proposed to aid in digestion through mechanical action, provide mineral supplementation, maintain hydrostatic balance, and inoculate the gut with microorganisms (Sokol, 1971; Terebiznik *et al*., 2020). Hence, it may not be accurate to categorise this behaviour as true self‐medication, although there is a case to be made for its non‐dietary health‐maintenance properties.

A compelling example of non‐medicinal, but nonetheless medical behaviour of significant antiparasitic and general hygienic value, is the chafing displayed by fishes, supposedly aimed at dislodging external parasites and alleviating skin irritation (Grossman, Sazima & Sazima, 2009; Berthe, Lecchini & Mourier, 2017; Thompson & Meeuwig, 2022; Williams *et al*., 2022). In most cases, fishes utilise inanimate objects such as sand or rocks with rough surfaces to rub against (Ritter, 2011; Berthe *et al*., 2017). However, in environments lacking such structures (e.g. for pelagic fishes), fishes engage in interspecific chafing, rubbing against turtles, sharks and other fishes (Grossman *et al*., 2009; Thompson & Meeuwig, 2022; Williams *et al*., 2022). The abrasive, sandpaper‐like texture of sharks' dermal denticles appears particularly well suited for this purpose (Papastamatiou, Meyer & Maragos, 2007; Thompson & Meeuwig, 2022), and provides an example of interspecific grooming behaviour.

Behavioural fever, an adaptive response observed across all classes of ectothermic vertebrates (see review by Rakus, Ronsmans & Vanderplasschen, 2017), exemplifies a more nuanced candidate of medical behaviour. Here, animals actively seek out warm environments (e.g. basking in sunlight) to elevate their core body temperature beyond its normal range, presumably aiming to combat infection (Rakus *et al*., 2017; Sauer *et al*., 2019). Analogous to endothermic vertebrates, raising body temperature promotes self‐healing by stimulating and providing optimal conditions for the immune system as well as negatively affecting pathogens (Merchant *et al*., 2007; Rakus *et al*., 2017; Sauer *et al*., 2019). However, unlike endotherms (which express physiological fever), ectotherms achieve febrile temperatures through behavioural regulation, namely thermoregulatory (movement) behaviour. Behavioural fever in reptiles and amphibians has been predominantly studied under laboratory conditions, revealing its induction by bacteria, viruses, fungi, and ectoparasites (see review by Rakus *et al*., 2017). Sauer *et al*. (2019) demonstrated behavioural fever in response to ranavirus inoculation in both adult and metamorphic southern toads (*Anaxyrus terrestris*).

Finally, one example drawn from the folklore of ancient Brazilian communities suggests self‐medication in lizards, where these animals appear to utilise plants for their therapeutic properties. It is said that lizards of the genus *Tupinambis* can counteract the effects of lethal snake bites by consuming the root of a specific plant with alleged anti‐venomous properties (Costa‐Neto, 2012; Shurkin, 2014). Similar folktales exist for snakes themselves and mongoose that are known to attack cobra (Huffman, 2022).

## III. INVERTEBRATES

It is well established that certain invertebrates utilise secondary plant compounds for defensive purposes (Boppré, 1984; Pawlik *et al*., 1988; Hunter, 2003; Ode, 2006; Opitz & Muller, 2009; Beran & Petschenka, 2022) and that the susceptibility of various herbivorous insects to pathogens depends on the plants on which they feed (Cory & Hoover, 2006). Recent insights have challenged the popular assumptions that medication behaviour necessitates advanced cognitive abilities, revealing that it occurs in animals such as insects where it is taxonomically widespread (Lefèvre *et al*., 2010; Abbott, 2014; de Roode & Hunter, 2019; Erler *et al*., 2024). The discovery of allo‐ and self‐medication behaviours in insects, exemplified by flies, butterflies, ants, and bees, has substantially expanded our previous understanding of self‐medication (de Roode & Hunter, 2019; Erler *et al*., 2024).

Early research conducted by Singer *et al*. (2009) on *Grammia incorrupta* (or *G. geneura*) caterpillars and their tachinid fly parasitoids has been highly influential. This study unequivocally demonstrated therapeutic self‐medication in an invertebrate for the first time, providing valuable insights into the innate and adaptive value of such behaviours. The dietary choice of individuals was determined by physiological plasticity, with caterpillars demonstrating a preference for protective secondary plant compounds (pyrrolizidine alkaloids) when parasitised, without the need for associative learning (Bernays & Singer, 2005; Singer *et al*., 2009). The revelation that *G. incorrupta* caterpillars self‐medicate by increasing the intake of components present in their natural diet showed that self‐medication in these insects can be quantitative and qualitative (Singer *et al*., 2009; de Roode & Hunter, 2019).

Research on insects has facilitated the adoption of an adaptive plasticity framework. While determining the costs of medication behaviour in vertebrates is challenging, such costs are relatively straightforward to quantify in insects through experimental manipulation (de Roode *et al*., 2013; de Roode & Hunter, 2019). Insects serve as an ideal animal model system for self‐medication studies due to the ease of experimental manipulation, offering several key advantages. Firstly, the ethical regulations pertaining to insect utilisation in scientific research are less complex than those governing vertebrate experimental subjects. Secondly, insect maintenance is more economically sustainable and creation of experimental groups (treated and untreated) is less complicated. Finally, the high reproductive rate and short lifespan of insects allow for many replicates and rapid observation of responses (Lefèvre *et al*., 2010; de Roode & Lefèvre, 2012; Abbott, 2014; Lucon‐Xiccato, Carere & Baracchi, 2024).

Research into insect self‐medication has yielded valuable insights into the social aspects of this behaviour, broadening its study within the context of inclusive fitness theory (Hamilton, 1964; Grafen, 1984; Hart, 1990; Lefèvre *et al*., 2010). For instance, investigation into the oviposition behaviour of monarch butterflies (*Danaus plexippus*) infected with the vertically transmitted protozoan parasite *Ophryocystis elektroscirrha* revealed a pattern of host‐plant selection based on secondary compound contents. This behaviour ultimately reduces parasite growth and disease in their offspring, marking the earliest documented instance of transgenerational therapeutic medication (Lefèvre *et al*., 2010, 2012; Tao *et al*., 2016). Such behaviours are unlikely to occur in vertebrate species, where parental care is an essential aspect of their biology.

Compared to other insect taxa, prophylactic self‐medication is perhaps most evident in eusocial Hymenoptera, such as ants and bees, where it is often directed towards genetic kin (Abbott, 2014; de Roode & Hunter, 2019). This behaviour contributes to social immunity by reducing infection risk at the colony level (Cremer, Armitage & Schmid‐Hempel, 2007; Cremer, Pull & Fürst, 2018). This research led to the term “social medication” for eusocial insects, signifying that the criteria for self‐medication extend beyond individual actions to encompassing colony‐level behaviours (Spivak, Goblirsch & Simone‐Finstrom, 2019). For example, wood ants (*Formica paralugubris*) actively incorporate antimicrobial resin from conifer trees into their nest, helping to prevent microbial growth and reducing reliance on their immune system (Christe *et al*., 2003; Chapuisat *et al*., 2007; Castella, Chapuisat & Christe, 2008). The collection of antimicrobial resin by ants is triggered by the presence of offspring, rather than pathogens, thus characterising social prophylaxis (Castella *et al*., 2008; Brütsch & Chapuisat, 2014; Cremer *et al*., 2018). Furthermore, this behaviour exemplifies a case of defensive mixology whereby ants enhance the potency of collected resin, by combining it with endogenous formic acid, effectively creating an “antimicrobial cocktail” (Brütsch *et al*., 2017).

Comparable behaviour is observed in honey bees (*Apis mellifera*), which routinely construct “in‐hive pharmacies” of propolis, nectar, pollen, royal jelly and beeswax (Erler *&* Moritz, 2016). One aspect receiving particular attention is the use of propolis obtained by combining antimicrobial resin with beeswax (Simone, Evans & Spivak, 2009; Simone‐Finstrom & Spivak, 2010; Simone‐Finstrom *et al*., 2017). Resin collection and propolis accumulation markedly increase during parasite infestation, suggesting prophylactic and therapeutic social medication (Simone‐Finstrom & Spivak, 2012; Pusceddu *et al*., 2019). In addition to its antimicrobial properties, propolis enhances the tolerance of honey bees to *Varroa* mite infection (Pusceddu *et al*., 2018) and interferes with viruses transmitted by these mites (Drescher *et al*., 2017). Furthermore, infested colonies benefit from increased levels of resin, given its lifespan‐extending properties in *Varroa*‐infested adults and its negative effects on parasite fitness, including narcolepsy and fertility reduction (Pusceddu *et al*., 2018, 2021a).

Immunisation has been described in vertebrates (Hart, 1990, 2011), ants (Stroeymeyt *et al*., 2018), and bees (Salmela, Amdam & Freitak, 2015; Harwood *et al*., 2021). In a honey bee hive, the queen transfers pathogen fragments to eggs, where they are recognised by the embryo's immune system and induce enhanced pathogen resistance in the new offspring (Salmela *et al*., 2015). These pathogen fragments are transported by vitellogenin (Vg), an egg‐yolk precursor protein that nurse bees also partially utilise to synthesise royal jelly. Consequently, royal jelly may serve as a vehicle for transporting pathogen fragments to larvae for up to 3 days. When these pathogenic cells are incorporated into royal jelly and consumed, the levels of an antimicrobial peptide present in royal jelly (defensin‐1) increase, thus it likely functions partially as a vaccination booster (Harwood *et al*., 2021).

Another prevalent manifestation of social immunity includes medical behaviours such as allo‐grooming and sanitary brood care, as observed in ants, bees, wasps, and termites. This behaviour consists of colony members removing ectoparasites from their genetic kin or ejecting infected brood from the colony (Kuswadi, 1992; Hughes, Eilenberg & Boomsma, 2002; Wilson‐Rich *et al*., 2009; de Roode & Lefèvre, 2012; Cini *et al*., 2020). Furthermore, eusocial insects, such as honey bees, perform social distancing behaviour to adapt their social and spatial distribution according to the infection status of the colony, aiming to reduce pathogen spread (Stroeymeyt *et al*., 2018; Stockmaier *et al*., 2021). For instance, Pusceddu *et al*. (2021b) revealed an increase in allo‐grooming bees in the hive's most vulnerable areas in the presence of *Varroa* mites. An intriguing adaptation of grooming behaviour is seen in garden ants (*Lasius neglectus*) that combine physical grooming with endogenous chemicals to remove and neutralise fungal spores from brood. These ants orally collect formic acid‐containing venom through self‐grooming and subsequently apply this antifungal treatment to the brood (Tragust *et al*., 2013). Additionally, these ants spray venom onto pupae (Tragust *et al*., 2013) and around the nest (Brütsch *et al*., 2017; Pull *et al*., 2018), while fire ant queens (*Solenopsis invicta*) apply endogenous antimicrobial and antifungal venom to their eggs during oviposition (Vander Meer & Morel, 1995). Lastly, it should be noted that grooming behaviour extends beyond eusocial taxa (de Roode & Lefèvre, 2012); it has been observed in damselflies (*Ischnura verticalis*), which use their claws to remove mites (Leung, Forbes & Baker, 2001).

A complex medical behaviour has been demonstrated in ants of the species *Megaponera analis* that conduct raids on termite nests, during which some ants are injured by termite soldiers. Researchers observed that after raids, healthy ants would carry injured nest mates back to the nest. Injured ants left in the field had a 32% mortality rate, whereas those carried back to the nest had a mortality rate close to 0% (Frank *et al*., 2017). This represents a form of rescue behaviour in invertebrates, focusing on injured individuals rather than on those in imminent danger (Frank *et al*., 2017), but this study did not provide any information about how the injured individuals brought back to the colony were treated. It has been suggested that this behaviour evolved due to specific factors in this species' ecology, including group hunting of dangerous prey and the high value of individual ants to the colony (Frank *et al*., 2017). Another intriguing aspect in rescue behaviour has recently been demonstrated. *Camponotus floridanus* worker ants have been shown to amputate the legs of nestmates with femur injuries, but not tibia injuries (Frank *et al*., 2024). Amputation of femur‐injured legs significantly increased survival and reduced pathogen load compared to untreated ants, while, for tibia injuries, amputation did not improve survival (Frank *et al*., 2024). This is the first demonstration of amputation in a non‐human animal to improve the survival of an injured conspecific. These two studies provide new insights into the evolution of helping and medical behaviours in animals, and the sophisticated adaptations of social insects.

Social fever represents a unique form of (behavioural) fever seen in honey bees, to combat heat‐sensitive pathogens within the social context of the hive (Starks, Blackie & Seeley, 2000; Goblirsch *et al*., 2020). Starks *et al*. (2000) demonstrated a 20% increase in brood‐comb temperature following experimental inoculation with the fungus *Ascosphaera apis*, indicating an adaptive, behavioural fever response. Behavioural fever in the classical sense is also observed in insects, limiting pathogen growth and proliferation, or resulting in pathogen death (Elliot, Blanford & Thomas, 2002; Ouedraogo *et al*., 2003; Ouedraogo, Goettel & Brodeur, 2004; Campbell *et al*., 2010; de Roode & Lefèvre, 2012; Zembrzuski *et al*., 2023). Alternatively, some insects, such as cockroaches (*Supella longipalpa* and *Blatta orientalis*) and bumble bees (*Bombus terrestris*), exhibit a behaviour known as “chilling”, by actively seeking out cold environments to impede parasite development and favour host survival (Müller & Schmid‐Hempel, 1993; Moore & Freehling, 2002).

Bumble bees exhibit various forms of medication behaviours. Recently, Figueroa *et al*. (2023) reported the mechanical effect of sunflower (*Helianthus annuus*) pollen in suppressing *Crithidia bombi*, a common gastrointestinal parasite of bumble bees (e.g. *Bombus impatiens* and *B. terrestris*). Like the leaf‐swallowing behaviour described in great apes and other mammals, the antiparasitic properties of sunflower pollen were attributed to its spiny exterior (Figueroa *et al*., 2023). Although it remains unclear whether bumble bees actively seek out sunflowers when infected, *Nosema‐*infected honey bees do exhibit a preference for sunflower honey, known for its antiparasitic effects (Gherman *et al*., 2014; Giacomini *et al*., 2018). Additionally, bumble bees infected with *C. bombi* display an increased inclination towards floral nectar containing antiparasitic compounds (alkaloids), suggesting self‐medication (Manson, Otterstatter & Thomson, 2010; Richardson *et al*., 2015). Another study demonstrated a prophylactic effect of heather (*Calluna vulgaris*) nectar consumption on *C. bombi* infectivity, attributed to secondary compounds that interfere with parasite attachment in the gut (Koch *et al*., 2019). Interestingly, Lepidoptera caterpillars ingest nicotine‐rich tobacco leaves and many other toxic plants when infected by parasitoid wasps (Quicke, Ghafouri Moghaddam & Butcher, 2023).

Although there is ample evidence of medical and medication strategies among insect taxa, such behaviours remain largely unexplored in other invertebrates. Future investigations may reveal instances in non‐insect species, as opportunities for such behaviours undoubtedly occur. In particular, the aquatic environment remains almost unexplored despite the increasing recognition of the medicinal properties of micro‐ and macroalgae for example (Korzeniowska *et al*., 2018; Sánchez *et al*., 2019; Dai *et al*., 2022). For instance, the Spanish dancer (*Hexabranchus sanguineus*) nudibranch sequesters toxins from its sponge diet as a defence mechanism against predators and parasites (Pawlik *et al*., 1988). Similarly to venom dart frogs (Dendrobatidae), these sea slugs incorporate sequestered toxins into their eggs, assumed to offer protection against pathogens, thus possibly representing a case of prophylactic medication (Pawlik *et al*., 1988). Maternal egg provisioning of tetrodotoxin by blue‐ringed octopuses (*Hapalochlaena* spp.) has also been documented (Williams *et al*., 2011). Although the exact source of this toxin in these species remains debated, evidence suggests an exogenous bacterial origin (Hwang *et al*., 1989). Identifying medical or medication behaviours in aquatic animals presents unique challenges given the extensive time required underwater for observing such behaviour in the wild, but this should not deter us from further investigation.

## IV. DISCUSSION

This comparative review draws upon different examples from diverse taxa to illustrate our growing comprehension of animal medical systems, using both medicinal substances and behaviours of medical value. It demonstrates the prevalence and adaptive significance of such systems across many animal taxa. From *Apis* to apes, remarkable adaptations to parasitism are revealed, unveiling myriad strategies employed by organisms to maintain health and fitness. These include behaviours such as geophagy, fur rubbing, anting, nest fumigation, behavioural fever, rescue behaviour, and maternal provisioning. Such behaviours demonstrate striking similarities, and sometimes differences, in how animals respond to health challenges, regardless of their cognitive capacity, revealing their widespread occurrence across taxa and niches. Understanding the prevalence and adaptive significance of each behaviour provides valuable insights into the evolutionary and ecological dynamics shaping animal health and survival strategies.

Among non‐medicative behaviours, grooming has many functions across a diverse range of species (Sachs, 1988; Spruijt, Van Hooff & Gispen, 1992). From primates meticulously picking through fur to remove lice eggs, to insects grooming themselves to remove ectoparasites, grooming is fundamental to animal hygiene and health maintenance (Sachs, 1988; Tanaka, 1995; Zamma, 2002). Only in vertebrates does grooming behaviour assume a significant social role, and may be exchanged for agonistic support or infant handling (Schino, 2001, 2007; Lazaro‐Perea, Arruda & Snowdon, 2004; Koyama, Caws & Aureli, 2006; Tiddi, Aureli & Schino, 2012; Caselli *et al*., 2021). Similarly, the ingestion of specific substances with medicinal properties, such as plants or soil, is widespread. These behaviours have been observed in diverse species ranging from primates to insects, but their common purpose remains consistent: combating parasites or absorbing infection/dietary toxins (Jain *et al*., 2008; Mamillapalli *et al*., 2016; de Roode & Hunter, 2019; Pebsworth *et al*., 2019; De la Fuente *et al*., 2022). Thermoregulatory behaviours, such as fever induction, represent adaptive defence mechanisms observed across taxa (Hart, 1990; Rakus *et al*., 2017).

The prevalence of these common medical strategies suggests ancient traits conserved throughout evolution (Sachs, 1988). Furthermore, the occurrence of behavioural fever in neonatal mammals unable to develop physiological fever (Rakus *et al*., 2017) indicates that it may represent an ancestral mechanism to combat illness. This is also exemplified by the sunbathing (thermoregulatory) behaviour of birds basking in direct sunlight as a possible mechanism to combat parasites (Bush & Clayton, 2018). Ancestral behaviours are likely to have been modified and refined in response to specific ecological challenges faced by different species in their respective habitats. For instance, although grooming behaviour may have originated as a means of cleaning oneself and removing ectoparasites (Sachs, 1988), it can be adapted to include the application of medicinal substances, as observed in orangutans (Morrogh‐Bernard *et al*., 2017; Laumer *et al*., 2024), and for external immunity in social insects (Tragust *et al*., 2013; Otti, Tragust & Feldhaar, 2014). Similarly, the alternative functions of soil, clay, dirt, or rock consumption by reptiles and amphibians (Sokol, 1971; Jain *et al*., 2008; Mamillapalli *et al*., 2016; Terebiznik *et al*., 2020) may reflect the evolutionary origins of geophagy. Such a behaviour may have emerged to maintain physiological homeostasis, and later became adapted into a specific set of behaviours to mitigate dietary toxicity or disease symptoms, highlighting the adaptive flexibility of medicinal strategies.

The presence of analogous behaviours across social and solitary species further reinforces the adaptive flexibility of medicinal strategies. Grooming behaviour exhibits similar functions in both social and solitary species (Sachs, 1988). Moreover, fever occurs in social models of animal medical behaviours, as evidenced by the expression of social fever in eusocial Hymenoptera (Hart, 1990; Starks *et al*., 2000). This response exemplifies convergent evolution between these invertebrate “superorganisms” and other fever‐producing animals (Starks *et al*., 2000). However, social fever differs from behavioural fever observed in other ectotherms, as it is attained through endogenous heat production (de Villepin, 2023), rendering it more akin to physiological fever.

The specific ecological challenges faced by different populations of the same species also shape the expression of animal medical systems (Hart, 1990; MacIntosh & Huffman, 2010; McLennan & Huffman, 2012; Tasdemir *et al*., 2020). This is exemplified by the vast array of antiparasitic behaviours of primates (Huffman, 2016; De la Fuente *et al*., 2022). Species in habitats with conditions of high parasite presence may exhibit heightened medication behaviours for survival (McLennan & Huffman, 2012; Choisy & de Roode, 2014; de Roode & Hunter, 2019; Viviano *et al*., 2022). The striking similarity between the use of spiny sunflower pollen by bumble bees, which is suggested to combat intestinal parasites mechanically (Figueroa *et al*., 2023), and the leaf‐swallowing behaviour of African great apes, Chinese lesser civets, brown bears, and snow geese (Huffman, 1997; Su *et al*., 2013) likely represents another case of convergent evolution where multiple taxa use resources within their immediate environment to deal with a health issue. Consequently, certain medicinal behaviours likely emerged independently across different taxa as adaptive responses to ecological challenges, illustrating the intricate relationship between organisms and their environment. In parallel, behavioural flexibility is evidenced among individuals of the same species who show similar behaviours in very different eco‐climatic habitats in response to different parasites (Huffman *et al*., 2009).

Sociality, or the degree of interaction among individuals within a social group, can significantly influence the prevalence and diversity of behavioural defence strategies. In social species, the risk of disease and parasite transmission is often heightened due to increased contact among individuals (Cremer *et al*., 2007, 2018). Consequently, social animals tend to express a broader spectrum of medical and medication behaviours, both individually and collectively (de Roode & Lefèvre, 2012). For example, social grooming behaviours in primates serve to maintain general hygiene and reduce the burden of body lice by regularly removing lice eggs (Huffman, 1997; Bowler *et al*., 2015; Morozov, 2015; Wilson *et al*., 2020). Nest fumigation is another example of medication in vertebrates with antiparasitic effects within a social context (Wimberger, 1984; Bush & Clayton, 2018). Similarly, social insect colonies engage in collective behaviours such as allo‐grooming, sanitary brood care, and incorporation of antimicrobial materials into their nests to combat pathogens and parasites (Castella *et al*., 2008; de Roode & Lefèvre, 2012; Tragust *et al*., 2013).

Solitary species may manifest more specialised forms of these behaviours, tailored to their specific ecological niches and individual requirements (Makuya & Schradin, 2024). For instance, in reptiles, amphibians and invertebrates, phenomena such as sequestration and maternal provisioning of dietary toxins (Pawlik *et al*., 1988; Williams *et al*., 2011; Stynoski *et al*., 2014, 2014; Brooks *et al*., 2022; Villanueva *et al*., 2022) illustrate innovative adaptations to exploit noxious substances inherent to their ecological context, benefiting either themselves or their offspring. The same argument can be made for the self‐medicating behaviour expressed by butterflies. These examples highlight the significant role played by ecological niches in shaping medicinal patterns across various animal taxa among invertebrates and vertebrates. Furthermore, these patterns suggest that life‐history traits, such as sociality and ecology, may be stronger predictors of the kind of medicinal behaviours manifested than cognitive abilities.

The relationship between cognition and medical strategies in animals is complex and multifaceted. Whereas cognitive abilities do contribute to certain medication and medical behaviours (e.g. problem‐solving and learning), their influence varies across different animal taxa. Particularly, the role of learning from conspecifics significantly influences the manifestation of self‐medication in social species, where individuals may have ample opportunities for close contact with an individual expert in the behaviour (Huffman & Hirata, 2004; Huffman *et al*., 2010). For example, plant‐processing behaviours and resource‐location knowledge recognised in great apes, as exemplified by leaf‐swallowing behaviour and bitter pith chewing, are believed to be socially transmitted (Huffman & Hirata, 2004), allowing such knowledge to spread reliably within the group. Another example is the recently acknowledged role of learning in various intricate behaviours of eusocial Hymenoptera, such as bumble bees (Chittka, 2017; Chittka & Rossi, 2022; Bridges *et al*., 2024). Therefore, the broader range of medication and medical behaviours in social species may also be attributed to the accumulation of learned knowledge maintained within social groups.

While social learning from conspecifics contributes to the acquisition of various aspects of medication and medical behaviours in social species, non‐social species also exhibit similar behaviours, which may be derived from innate tendencies and individual learning influenced by ecological pressures. However, for species that provide postnatal care, all young receive some level of information about the environment from their parents, at least until they are independent. Thus, parental care can play a crucial role in transmitting behaviours from one generation to the next, thereby maintaining them within a population. However, the occurrence of identical behaviours in geographically isolated species suggests the involvement of an innate, or phylogenetically conserved behavioural propensity, even in great apes and monkeys (Huffman & Hirata, 2003). The taxonomic and geographic ubiquity of anting and dust‐bathing in birds (Clayton *et al*., 2010; Morozov, 2015; Bush & Clayton, 2018) also indicates the involvement of an underlying phylogenetically conserved trait, with habitat‐specific information on the plant species used possibly maintained within populations by transmission of behaviours from parents to offspring. The innate and adaptive mechanisms behind various self‐medicating behaviours in solitary arthropods are discussed in detail by Singer *et al*. (2009).

Several examples suggest that individual learning plays a role in shaping self‐medication behaviours with an underlying innate basis. For instance, the refinement of anointing behaviour in orangutans, achieved through the galvanic preparation of *Dracaena cantleyi* leaf extracts to enhance their potency (Morrogh‐Bernard, 2008; Morrogh‐Bernard *et al*., 2017), is possibly the result of individual trial‐and‐error by a feedback learning process. Ants may have a similar mechanism that allows them to boost the antibacterial properties of resin (Brütsch *et al*., 2017). Inter‐site variation in fur‐rubbing material selection in capuchins is likely due to habitat differences in species availability, but the selection criteria for these materials is likely to be based on underlying innate olfactory responses to chemical cues. Animals incorporate different resources from their immediate environment, including plants, fruit, and arthropods (Baker, 1996; DeJoseph *et al*., 2002; Verderane *et al*., 2007; Medeiros *et al*., 2020) to increase the efficiency of such medicinal behaviour within their own ecological context. Thus, we hypothesise that innate behavioural propensities drive some aspects of medicinal behaviour in both social and non‐social species of invertebrates and vertebrates. Learning and innate responses likely contribute to refining and adapting animal medication to specific ecological contexts in a very wide variety of taxa.

## V. CONCLUSIONS

(1) An intricate interplay between innate predispositions and learned behaviours in response to variation in ecological context seems to shape the expression and diversity of medical systems across the animal kingdom.
(2) Both invertebrates and vertebrates can develop sophisticated medicinal strategies based on social and/or individual learning. In addition, life‐history traits emerge as strong predictors due to their direct impact on disease risk and ecological context.
(3) It is important to study diverse animal models to gain a comprehensive understanding of the mechanisms underlying medical behaviour and its adaptive significance in nature.
(4) Nevertheless, many questions remain unanswered. (*i*) Is the innate basis for these behaviours consistent across taxa, or did different behaviours evolve independently? (*ii*) Under what ecological and social conditions did medication first evolve, and are processes for maintaining physiological homeostasis fundamental to its most primitive forms? (*iii*) Is the prominence of medicinal behaviour in social species primarily due to knowledge transfer between conspecifics, is it driven by a greater need for antiparasitic defence, or have we simply been biased in our assumptions about where and how to look for it? (*iv*) What are the ecological implications of the emergence of these behaviours within populations? (*v*) To what extent do social dynamics within animal groups influence the transmission of medicinal behaviours, and to what extent does observational learning contribute to medicinal behaviour in non‐social species? (*vi*) Are there universal principles underlying medicinal behaviours, or do they vary significantly depending on environmental factors and species‐specific characteristics? (*vii*) Is parasitic pressure an evolutionary driver that has resulted in many forms of medicinal behaviour? Such questions open new avenues for research and highlight the intricate nature of medicinal and medical systems in the animal kingdom.

## References

[brv70060-bib-0001] Abbott, J. (2014). Self‐medication in insects: current evidence and future perspectives. Ecological Entomology 39(3), 273–280.

[brv70060-bib-0002] Alabssawy, A. N. (2017). Antimicrobial activity of tetrodotoxin extracted from liver, skin and muscles of puffer fish, *Lagocephalus sceleratus* inhabiting Mediterranean Sea, Egypt. International Journal of Cancer and Biomedical Research 1(1), 2–10.

[brv70060-bib-0003] Amit, M. , Cohen, I. , Marcovics, A. , Muklada, H. , Glasser, T. A. , Ungar, E. D. & Landau, S. Y. (2013). Self‐medication with tannin‐rich browse in goats infected with gastro‐intestinal nematodes. Veterinary Parasitology 198(3–4), 305–311. 24140164 10.1016/j.vetpar.2013.09.019

[brv70060-bib-0004] Arlettaz, R. , Christe, P. , Surai, P. F. & Møller, A. P. (2002). Deliberate rusty staining of plumage in the bearded vulture: does function precede art? Animal Behaviour 64(3), F1–F3.

[brv70060-bib-0005] Azaizeh, H. , Halahleh, F. , Abbas, N. , Markovics, A. , Muklada, H. , Ungar, E. D. & Landau, S. Y. (2012). Polyphenols from *Pistacia lentiscus* and *Phillyrea latifolia* impair the exsheathment of gastro‐intestinal nematode larvae. Veterinary Parasitology 191(1–2), 44–50. 22985927 10.1016/j.vetpar.2012.08.016

[brv70060-bib-0006] Baker, M. (1996). Fur rubbing: use of medicinal plants by capuchin monkeys (*Cebus capucinus*). American Journal of Primatology 38(4), 263–270.

[brv70060-bib-0007] Baker, M. (2017). Fur Rubbing. In The International Encyclopedia of Primatology (ed. A. Fuentes ). John Wiley & Sons, Inc., Hoboken, NJ.

[brv70060-bib-0008] Barelli, C. & Huffman, M. A. (2017). Leaf swallowing and parasite expulsion in Khao Yai white‐handed gibbons (*Hylobates lar*), the first report in an Asian ape species. American Journal of Primatology 79(3), e22610. 10.1002/ajp.22610 28118500

[brv70060-bib-0009] Beran, F. & Petschenka, G. (2022). Sequestration of plant defense compounds by insects: from mechanisms to insect–plant coevolution. Annual Review of Entomology 67(1), 163–180. 10.1146/annurev-ento-062821-062319 34995091

[brv70060-bib-0010] Bernays, E. A. & Singer, M. S. (2005). Insect defences: taste alteration and endoparasites. Nature 436(7050), 476. 16049466 10.1038/436476a

[brv70060-bib-0011] Berthe, C. , Lecchini, D. & Mourier, J. (2017). Chafing behavior on a patch of sandy bottom by ocellated eagle ray (*Aetobatus ocellatus*). Marine Biodiversity 47, 379–380.

[brv70060-bib-0012] Birkinshaw, C. R. (1999). Use of millipedes by black lemurs to anoint their bodies. Folia Primatologica 70(3), 170–171. 10.1159/000021691 10394067

[brv70060-bib-0013] Blaise, A. , Kiewra, D. , Chrząścik, K. , Selva, N. , Popiołek, M. & Sergiel, A. (2023). Anti‐parasitic function of tree‐rubbing behaviour in brown bears suggested by an in vitro test on a generalist ectoparasite. Journal of Zoology 319(4), 296–307.

[brv70060-bib-0014] Boppré, M. (1984). Redefining “pharmacophagy”. Journal of Chemical Ecology 10, 1151–1154. 24318856 10.1007/BF00987520

[brv70060-bib-0015] Borruso, L. , Checcucci, A. , Torti, V. , Correa, F. , Sandri, C. , Luise, D. , Cavani, L. , Modesto, M. , Spiezio, C. , Mimmo, T. , Cesco, S. , Di Vito, M. , Bugli, F. , Randrianarison, R. M. , Gamba, M. , *et al*. (2021). I like the way you eat it: lemur (*Indri indri*) gut mycobiome and geophagy. Microbial Ecology 82, 215–223. 33471174 10.1007/s00248-020-01677-5 PMC8282574

[brv70060-bib-0016] Bowler, M. , Messer, E. J. , Claidière, N. & Whiten, A. (2015). Mutual medication in capuchin monkeys–social anointing improves coverage of topically applied anti‐parasite medicines. Scientific Reports 5(1), 15030. 26456539 10.1038/srep15030 PMC4601033

[brv70060-bib-0017] Bracke, M. B. (2011). Review of wallowing in pigs: description of the behaviour and its motivational basis. Applied Animal Behaviour Science 132(1–2), 1–13.

[brv70060-bib-0018] Bridges, A. D. , Royka, A. , Wilson, T. , Lockwood, C. , Richter, J. , Juusola, M. & Chittka, L. (2024). Bumblebees socially learn behaviour too complex to innovate alone. Nature 627(8004), 572–578. 38448580 10.1038/s41586-024-07126-4 PMC10954542

[brv70060-bib-0019] Brooks, O. L. , James, J. J. & Saporito, R. A. (2022). Maternal chemical defenses predict offspring defenses in a dendrobatid poison frog. Oecologia 201(2), 385–396. 10.1007/s00442-023-05314-z 36637523

[brv70060-bib-0020] Brucker, R. M. , Baylor, C. M. , Walters, R. L. , Lauer, A. , Harris, R. N. & Minbiole, K. P. (2008a). The identification of 2, 4‐diacetylphloroglucinol as an antifungal metabolite produced by cutaneous bacteria of the salamander *Plethodon cinereus* . Journal of Chemical Ecology 34, 39–43. 18058176 10.1007/s10886-007-9352-8

[brv70060-bib-0021] Brucker, R. M. , Harris, R. N. , Schwantes, C. R. , Gallaher, T. N. , Flaherty, D. C. , Lam, B. A. & Minbiole, K. P. (2008b). Amphibian chemical defense: antifungal metabolites of the microsymbiont *Janthinobacterium lividum* on the salamander *Plethodon cinereus* . Journal of Chemical Ecology 34, 1422–1429. 18949519 10.1007/s10886-008-9555-7

[brv70060-bib-0022] Brütsch, T. & Chapuisat, M. (2014). Wood ants protect their brood with tree resin. Animal Behaviour 93, 157–161.

[brv70060-bib-0023] Brütsch, T. , Jaffuel, G. , Vallat, A. , Turlings, T. C. J. & Chapuisat, M. (2017). Wood ants produce a potent antimicrobial agent by applying formic acid on tree‐collected resin. Ecology and Evolution 7(7), 2249–2254. 28405288 10.1002/ece3.2834 PMC5383563

[brv70060-bib-0024] Bush, S. E. & Clayton, D. H. (2018). Anti‐parasite behaviour of birds. Philosophical Transactions of the Royal Society, B: Biological Sciences 373(1751), 20170196. 10.1098/rstb.2017.0196 PMC6000146 29866911

[brv70060-bib-0025] Campbell, J. , Kessler, B. , Mayack, C. & Naug, D. (2010). Behavioural fever in infected honeybees: parasitic manipulation or coincidental benefit? Parasitology 137(10), 1487–1491. 20500914 10.1017/S0031182010000235

[brv70060-bib-0026] Caselli, M. , Zanoli, A. , Palagi, E. & Norscia, I. (2021). Infant handling increases grooming towards mothers in wild geladas (*Theropithecus gelada*). Behavioural Processes 192, 104501. 34517089 10.1016/j.beproc.2021.104501

[brv70060-bib-0027] Castella, G. , Chapuisat, M. & Christe, P. (2008). Prophylaxis with resin in wood ants. Animal Behaviour 75(4), 1591–1596.

[brv70060-bib-0028] Chapuisat, M. , Oppliger, A. , Magliano, P. & Christe, P. (2007). Wood ants use resin to protect themselves against pathogens. Proceedings of the Royal Society B: Biological Sciences 274(1621), 2013–2017. 10.1098/rspb.2007.0531 PMC2275180 17535794

[brv70060-bib-0029] Chau, R. , Kalaitzis, J. A. & Neilan, B. A. (2011). On the origins and biosynthesis of tetrodotoxin. Aquatic Toxicology 104(1–2), 61–72. 21543051 10.1016/j.aquatox.2011.04.001

[brv70060-bib-0030] Chittka, L. (2017). Bee cognition. Current Biology 27(19), R1049–R1053. 29017035 10.1016/j.cub.2017.08.008

[brv70060-bib-0031] Chittka, L. & Rossi, N. (2022). Social cognition in insects. Trends in Cognitive Sciences 26(7), 578–579. 35570086 10.1016/j.tics.2022.04.001

[brv70060-bib-0032] Choisy, M. & de Roode, J. C. (2014). The ecology and evolution of animal medication: genetically fixed response versus phenotypic plasticity. The American Naturalist 184(S1), S31–S46. 10.1086/676928 25061676

[brv70060-bib-0033] Christe, P. , Oppliger, A. , Bancalà, F. , Castella, G. & Chapuisat, M. (2003). Evidence for collective medication in ants. Ecology Letters 6(1), 19–22.

[brv70060-bib-0034] Cini, A. , Bordoni, A. , Cappa, F. , Petrocelli, I. , Pitzalis, M. , Iovinella, I. , Dani, F. R. , Turillazzi, S. & Cervo, R. (2020). Increased immunocompetence and network centrality of allogroomer workers suggest a link between individual and social immunity in honeybees. Scientific Reports 10(1), 8928. 32488140 10.1038/s41598-020-65780-w PMC7265547

[brv70060-bib-0035] Clark, C. C. (1991). The nest protection hypothesis: the adaptive use of plant secondary compounds by European starlings. In Bird‐Parasite Interactions: Ecology, Evolution, and Behavior (eds J. E. Loye and M. Zuk ), pp. 205–221. Oxford University Press, Oxford.

[brv70060-bib-0036] Clark, L. & Mason, J. R. (1985). Use of nest material as insecticidal and anti‐pathogenic agents by the European starling. Oecologia 67, 169–176. 28311305 10.1007/BF00384280

[brv70060-bib-0037] Clark, L. & Mason, J. R. (1988). Effect of biologically active plants used as nest material and the derived benefit to starling nestlings. Oecologia 77, 174–180. 28310369 10.1007/BF00379183

[brv70060-bib-0038] Clayton, D. H. , Koop, J. A. , Harbison, C. W. , Moyer, B. R. & Bush, S. E. (2010). How birds combat ectoparasites. The Open Ornithology Journal 3, 41–71.

[brv70060-bib-0039] Clayton, D. H. & Wolfe, N. D. (1993). The adaptive significance of self‐medication. Trends in Ecology & Evolution 8, 60–63. 21236108 10.1016/0169-5347(93)90160-Q

[brv70060-bib-0040] Cooney, D. O. & Struhsaker, T. T. (1997). Adsorptive capacity of charcoals eaten by Zanzibar red colobus monkeys: implications for reducing dietary toxins. International Journal of Primatology 18, 235–246.

[brv70060-bib-0041] Coppedge, B. R. & Shaw, J. H. (2000). American bison (*Bison bison*) wallowing behavior and wallow formation on tallgrass prairie. Acta Theriologica 45(1), 103–110.

[brv70060-bib-0042] Cory, J. S. & Hoover, K. (2006). Plant‐mediated effects in insect–pathogen interactions. Trends in Ecology & Evolution 21(5), 278–286. 16697914 10.1016/j.tree.2006.02.005

[brv70060-bib-0043] Costa‐Neto, E. M. (2012). Zoopharmacognosy, the self‐medication behavior of animals. Interfaces Científicas‐Saúde e Ambiente 1(1), 61–72.

[brv70060-bib-0044] Cremer, S. , Armitage, S. A. & Schmid‐Hempel, P. (2007). Social immunity. Current Biology 17(16), R693–R702. 17714663 10.1016/j.cub.2007.06.008

[brv70060-bib-0045] Cremer, S. , Pull, C. D. & Fürst, M. A. (2018). Social immunity: emergence and evolution of colony‐level disease protection. Annual Review of Entomology 63(1), 105–123. 10.1146/annurev-ento-020117-043110 28945976

[brv70060-bib-0046] Dai, N. , Wang, Q. , Xu, B. & Chen, H. (2022). Remarkable natural biological resource of algae for medical applications. Frontiers in Marine Science 9, 912924.

[brv70060-bib-0047] De la Fuente, M. F. , Souto, A. , Albuquerque, U. P. & Schiel, N. (2022). Self‐medication in nonhuman primates: a systematic evaluation of the possible function of the use of medicinal plants. American Journal of Primatology 84(11), e23438. 36193566 10.1002/ajp.23438

[brv70060-bib-0048] de Roode, J. C. & Huffman, M. A. (2024). Animal medication. Current Biology 34(17), R808–R812. 39255760 10.1016/j.cub.2024.07.034

[brv70060-bib-0049] de Roode, J. C. & Hunter, M. D. (2019). Self‐medication in insects: when altered behaviors of infected insects are a defense instead of a parasite manipulation. Current Opinion in Insect Science 33, 1–6. 31358187 10.1016/j.cois.2018.12.001

[brv70060-bib-0050] de Roode, J. C. & Lefèvre, T. (2012). Behavioral immunity in insects. Insects 3(3), 789–820. 26466629 10.3390/insects3030789 PMC4553590

[brv70060-bib-0051] de Roode, J. C. , Lefèvre, T. & Hunter, M. D. (2013). Self‐medication in animals. Science 340(6129), 150–151. 23580516 10.1126/science.1235824

[brv70060-bib-0052] de Villepin, F. E. (2023). Thermoregulation in honey bees. Part 1. American Bee Journal 163(12), 1299–1302.

[brv70060-bib-0053] DeJoseph, M. , Taylor, R. S. L. , Baker, M. & Aregullin, M. (2002). Fur‐rubbing behavior of capuchin monkeys. Journal of the American Academy of Dermatology 46(6), 924–925. 12063492 10.1067/mjd.2002.119668

[brv70060-bib-0054] Downs, C. T. , Bredin, I. P. & Wragg, P. D. (2019). More than eating dirt: a review of avian geophagy. African Zoology 54(1), 1–19.

[brv70060-bib-0055] Drescher, N. , Klein, A.‐M. , Neumann, P. , Yanez, O. & Leonhardt, S. D. (2017). Inside honeybee hives: impact of natural propolis on the ectoparasitic mite *Varroa destructor* and viruses. Insects 8(1), 15. 28178181 10.3390/insects8010015 PMC5371943

[brv70060-bib-0056] Dumbacher, J. P. & Pruett‐Jones, S. (1996). Avian chemical defense. In Current Ornithology (ed. S. Koenig ), pp. 137–174. Springer, Boston, MA.

[brv70060-bib-0057] Elliot, S. L. , Blanford, S. & Thomas, M. B. (2002). Host–pathogen interactions in a varying environment: temperature, behavioural fever and fitness. Proceedings of the Royal Society of London, Series B: Biological Sciences 269(1500), 1599–1607. 10.1098/rspb.2002.2067 PMC1691072 12184830

[brv70060-bib-0058] Engel, C. (2002). Wild Health. How Animals Keep Themselves Well and What We Can Learn from Them, p. 276. Hughton & Mifflin, Boston.

[brv70060-bib-0059] Erler, S. , Cotter, S. C. , Freitak, D. , Koch, H. , Palmer‐Young, E. C. , de Roode, J. C. , Smilanich, A. M. & Lattorff, H. M. G. (2024). Insects' essential role in understanding and broadening animal medication. Trends in Parasitology 40(4), 338–349. 38443305 10.1016/j.pt.2024.02.003

[brv70060-bib-0060] Erler, S. & Moritz, R. F. A. (2016). Pharmacophagy and pharmacophory: mechanisms of self‐medication and disease prevention in the honeybee colony (*Apis mellifera*). Apidologie 47, 389–411.

[brv70060-bib-0061] Ferrell, R. E. Jr. (2008). Medicinal clay and spiritual healing. Clays and Clay Minerals 56(6), 751–760.

[brv70060-bib-0062] Figueroa, L. L. , Fowler, A. , Lopez, S. , Amaral, V. E. , Koch, H. , Stevenson, P. C. , Irwin, R. E. & Adler, L. S. (2023). Sunflower spines and beyond: mechanisms and breadth of pollen that reduce gut pathogen infection in the common eastern bumble bee. Functional Ecology 37(6), 1757–1769.

[brv70060-bib-0063] Fischer, E. K. , Roland, A. B. , Moskowitz, N. A. , Vidoudez, C. , Ranaivorazo, N. , Tapia, E. E. , Trauger, S. A. , Vences, M. , Coloma, L. A. & O'Connell, L. A. (2019). Mechanisms of convergent egg provisioning in poison frogs. Current Biology 29(23), 4145–4151. 31761700 10.1016/j.cub.2019.10.032

[brv70060-bib-0064] Forbey, J. , Harvey, A. , Huffman, M. A. , Provenza, F. , Sullivan, R. & Tasdemir, D. (2009). Exploitation of secondary metabolites by animals: a behavioral response to homeostatic challenges. Integrative and Comparative Biology 49(3), 314–328. 21665822 10.1093/icb/icp046

[brv70060-bib-0065] Frank, E. T. , Buffat, D. , Liberti, J. & Keller, L. (2024). Wound‐dependent leg amputations to combat infections in an ant society. Current Biology 34(14), 3273–3278. 38959879 10.1016/j.cub.2024.06.021

[brv70060-bib-0066] Frank, E. T. , Schmitt, T. , Hovestadt, T. , Mitesser, O. , Stiegler, J. & Linsenmair, K. E. (2017). Saving the injured: rescue behavior in the termite‐hunting ant *Megaponera analis* . Science Advances 3(4), e1602187. 28439543 10.1126/sciadv.1602187 PMC5389746

[brv70060-bib-0067] Gall, B. G. , Stokes, A. N. , French, S. S. , Schlepphorst, E. A. , Brodie, E. D. III & Brodie, E. D. Jr. (2011). Tetrodotoxin levels in larval and metamorphosed newts (*Taricha granulosa*) and palatability to predatory dragonflies. Toxicon 57(7–8), 978–983. 21459104 10.1016/j.toxicon.2011.03.020

[brv70060-bib-0068] Gall, B. G. , Stokes, A. N. , French, S. S. , Schlepphorst, E. A. , Groffen, J. & Brodie, E. D. Jr. (2022). Antipredator defenses of Taricha newts: are toxic amphibians immune to snake predation? Journal of Zoology 318(2), 84–92.

[brv70060-bib-0069] Gherman, B. I. , Denner, A. , Bobiş, O. , Dezmirean, D. S. , Mărghitaş, L. A. , Schlüns, H. , Moritza, R. F. A. & Erler, S. (2014). Pathogen‐associated self‐medication behavior in the honeybee *Apis mellifera* . Behavioral Ecology and Sociobiology 68, 1777–1784.

[brv70060-bib-0070] Giacomini, J. J. , Leslie, J. , Tarpy, D. R. , Palmer‐Young, E. C. , Irwin, R. E. & Adler, L. S. (2018). Medicinal value of sunflower pollen against bee pathogens. Scientific Reports 8(1), 14394. 30258066 10.1038/s41598-018-32681-y PMC6158195

[brv70060-bib-0071] Glander, K. E. (1994). Nonhuman primate self‐medication with wild plant foods. In Eating on the Wild Side: The Pharmacologic, Ecologic, and Social Implications of Using Noncultigens (ed. N. L. Etkin ), pp. 227–239. The University of Arizona Press, Tucson.

[brv70060-bib-0072] Glasser, T. A. , Ungar, E. D. , Landau, S. Y. , Perevolotsky, A. , Muklada, H. & Walker, J. W. (2009). Breed and maternal effects on the intake of tannin‐rich browse by juvenile domestic goats (*Capra hircus*). Applied Animal Behaviour Science 119(1–2), 71–77.

[brv70060-bib-0073] Goblirsch, M. , Warner, J. F. , Sommerfeldt, B. A. & Spivak, M. (2020). Social fever or general immune response? Revisiting an example of social immunity in honey bees. Insects 11(8), 528. 32823597 10.3390/insects11080528 PMC7469213

[brv70060-bib-0074] Gompper, M. E. & Holyman, A. M. (1993). Grooming with *Trattinnickia* resin: possible pharmaceutical plant use by coatis in Panama. Journal of Tropical Ecology 9(4), 533–540.

[brv70060-bib-0075] Grafen, A. (1984). Natural selection, kin selection and group selection. In Behavioural Ecology, 2nd Edition (eds J. R. Krebs and N. B. Davies ), pp. 62–84. Blackwell Scientific Publications, Oxford.

[brv70060-bib-0076] Grant, T. , Colombo, P. , Verrastro, L. & Saporito, R. A. (2012). The occurrence of defensive alkaloids in non‐integumentary tissues of the Brazilian red‐belly toad *Melanophryniscus simplex* (Bufonidae). Chemoecology 22, 169–178.

[brv70060-bib-0077] Grossman, A. , Sazima, C. & Sazima, I. (2009). Rub and move: barracudas (*Sphyraena barracuda*) use swimming turtles as scraping surfaces in the South‐western Atlantic. Marine Biodiversity Records 2, e106.

[brv70060-bib-0078] Hamilton, W. D. (1964). The genetical evolution of social behaviour. I. Journal of Theoretical Biology 7(1), 1–16. 5875341 10.1016/0022-5193(64)90038-4

[brv70060-bib-0079] Hart, B. L. (1990). Behavioral adaptations to pathogens and parasites: five strategies. Neuroscience & Biobehavioral Reviews 14(3), 273–294. 2234607 10.1016/s0149-7634(05)80038-7

[brv70060-bib-0080] Hart, B. L. (2011). Behavioural defences in animals against pathogens and parasites: parallels with the pillars of medicine in humans. Philosophical Transactions of the Royal Society, B: Biological Sciences 366(1583), 3406–3417. 10.1098/rstb.2011.0092 PMC3189355 22042917

[brv70060-bib-0081] Harwood, G. , Salmela, H. , Freitak, D. & Amdam, G. (2021). Social immunity in honey bees: royal jelly as a vehicle in transferring bacterial pathogen fragments between nestmates. Journal of Experimental Biology 224(7), jeb231076. 10.1242/jeb.231076 34424968

[brv70060-bib-0082] Hayes, R. A. , Crossland, M. R. , Hagman, M. , Capon, R. J. & Shine, R. (2009). Ontogenetic variation in the chemical defenses of cane toads (*Bufo marinus*): toxin profiles and effects on predators. Journal of Chemical Ecology 35(4), 391–399. 19263169 10.1007/s10886-009-9608-6

[brv70060-bib-0083] Hemmes, R. B. , Alvarado, A. & Hart, B. L. (2002). Use of California bay foliage by wood rats for possible fumigation of nest‐borne ectoparasites. Behavioral Ecology 13(3), 381–385.

[brv70060-bib-0084] Hovey, K. J. , Seiter, E. M. , Johnson, E. E. & Saporito, R. A. (2018). Sequestered alkaloid defenses in the dendrobatid poison frog *Oophaga pumilio* provide variable protection from microbial pathogens. Journal of Chemical Ecology 44, 312–325. 29427191 10.1007/s10886-018-0930-8

[brv70060-bib-0085] Huffman, M. A. (1997). Current evidence for self‐medication in primates: a multidisciplinary perspective. American Journal of Physical Anthropology 104(S25), 171–200.

[brv70060-bib-0086] Huffman, M. A. (2003). Animal self‐medication and ethno‐medicine: exploration and exploitation of the medicinal properties of plants. Proceedings of the Nutrition Society 62(2), 371–381. 14506884 10.1079/pns2003257

[brv70060-bib-0087] Huffman, M. A. (2016). Primate self‐medication, passive prevention and active treatment‐a brief review. International Journal of Multidisciplinary Studies 3(2), 1–10.

[brv70060-bib-0088] Huffman, M. A. (2019). Self‐medication: passive prevention and active treatment. In Encyclopedia of Animal Behavior, 2nd Edition (Volume 2, ed. J. C. Choe ), pp. 696–702. Elsevier Academic Press, New York.

[brv70060-bib-0089] Huffman, M. A. (2022). Folklore, animal self‐medication, and phytotherapy–something old, something new, something borrowed, some things true. Planta Medica 88(3–4), 187–199. 34624907 10.1055/a-1586-1665

[brv70060-bib-0090] Huffman, M. A. & Caton, J. M. (2001). Self‐induced increase of gut motility and the control of parasitic infections in wild chimpanzees. International Journal of Primatology 22(3), 329–346.

[brv70060-bib-0091] Huffman, M. A. , Gotoh, S. , Izutsu, D. , Koshimizu, K. & Kalunde, M. S. (1993). Further observations on the use of the medicinal plant, *Vernonia amygdalina* (Del) by a wild chimpanzee, its possible effect on parasite load, and its phytochemistry. African Study Monographs 14(4), 227–240.

[brv70060-bib-0092] Huffman, M. A. & Hirata, S. (2003). Biological and ecological foundations of primate behavioral traditions. In The Biology of Traditions (eds D. M. Fragaszy and S. Perry ), pp. 267–296. University of Cambridge Press, Cambridge.

[brv70060-bib-0093] Huffman, M. A. & Hirata, S. (2004). An experimental study of leaf swallowing in captive chimpanzees: insights into the origin of a self‐medicative behavior and the role of social learning. Primates 45(2), 113–118. 15095043 10.1007/s10329-003-0065-5

[brv70060-bib-0094] Huffman, M. A. , Page, J. E. , Sukhdeo, M. V. , Gotoh, S. , Kalunde, M. S. , Chandrasiri, T. & Towers, G. N. (1996). Leaf‐swallowing by chimpanzees: a behavioral adaptation for the control of strongyle nematode infections. International Journal of Primatology 17, 475–503.

[brv70060-bib-0095] Huffman, M. A. , Pebsworth, P. , Bakuneeta, C. , Gotoh, S. & Bardi, M. (2009). Macro‐habitat comparison of host‐parasite ecology in two populations of chimpanzees in the Budongo forest, Uganda and the Mahale Mountains, Tanzania. In Primate Parasite Ecology: The Dynamics of Host‐Parasite Relationships (eds M. A. Huffman and C. Chapman ), pp. 311–330. Cambridge University Press, Cambridge.

[brv70060-bib-0096] Huffman, M. A. & Seifu, M. (1989). Observations on the illness and consumption of a possibly medicinal plant *Vernonia amygdalina* (Del.), by a wild chimpanzee in the Mahale Mountains National Park, Tanzania. Primates 30(1), 51–63.

[brv70060-bib-0097] Huffman, M. A. , Spiezio, C. , Sgaravatti, A. & Leca, J. B. (2010). Option biased learning involved in the acquisition and transmission of leaf swallowing behavior in chimpanzees (*Pan troglodytes*). Animal Cognition 13(6), 871–880. 20602132 10.1007/s10071-010-0335-8

[brv70060-bib-0098] Huffman, M. A. & Wrangham, R. W. (1994). Diversity of medicinal plant use by chimpanzees in the wild. In Chimpanzee Cultures (eds R. W. Wrangham , W. C. McGrew , F. B. M. de Waal and P. G. Heltne ), pp. 129–148. Harvard University Press, Cambridge.

[brv70060-bib-0099] Hughes, W. O. H. , Eilenberg, J. & Boomsma, J. J. (2002). Trade‐offs in group living: transmission and disease resistance in leaf‐cutting ants. Proceedings of the Biological Sciences 269(1502), 1811–1819. 12350269 10.1098/rspb.2002.2113 PMC1691100

[brv70060-bib-0100] Hunter, M. D. (2003). Effects of plant quality on the population ecology of parasitoids. Agricultural and Forest Entomology 5(1), 1–8.

[brv70060-bib-0101] Hutchings, M. R. , Athanasiadou, S. , Kyriazakis, I. , Gordon, I. J. & Pemberton, J. M. (2003). Can animals use foraging behavior to combat parasites? Proceedings of the Nutrition Society 62(2), 361–370. 14506883 10.1079/pns2003243

[brv70060-bib-0102] Hutchinson, D. A. , Savitzky, A. H. , Mori, A. , Meinwald, J. & Schroeder, F. C. (2007). Maternal provisioning of sequestered defensive steroids by the Asian snake *Rhabdophis tigrinus* . Chemoecology 18, 181–190.

[brv70060-bib-0103] Hwang, D. F. , Arakawa, O. , Saito, T. , Noguchi, T. , Simidu, U. , Tsukamoto, K. , Shida, Y. & Hashimoto, K. (1989). Tetrodotoxin‐producing bacteria from the blue‐ringed octopus *Octopus maculosus* . Marine Biology 100, 327–332.

[brv70060-bib-0104] Itoi, S. , Ueda, H. , Yamada, R. , Takei, M. , Sato, T. , Oshikiri, S. , Wajima, Y. , Ogata, R. , Oyama, H. , Shitto, T. , Okuhara, K. , Tsunashima, T. , Sawayama, E. & Sugita, H. (2018). Including planocerid flatworms in the diet effectively toxifies the pufferfish, Takifugu niphobles. Scientific Reports 8(1), 12302. 30120305 10.1038/s41598-018-30696-z PMC6098040

[brv70060-bib-0105] Jain, C. P. , Dashora, A. , Garg, R. , Kataria, U. & Vashistha, B. (2008). Animal self‐medication through natural sources. Natural Product Radiance 7(1), 49–53.

[brv70060-bib-0106] Janzen, D. H. (1978). Complications in interpreting the chemical defenses of trees against tropical arboreal plant‐eating vertebrates. In The Ecology of Arboreal Foliovores (ed. G. C. Montgomerie ), pp. 73–84. Smithsonian Institution Press, Washington, DC.

[brv70060-bib-0107] Johnson, P. T. J. , Calhoun, D. M. , Stokes, A. N. , Susbilla, C. B. , McDevitt‐Galles, T. , Briggs, C. J. , Hoverman, J. T. , Tkach, V. V. & de Roode, J. C. (2018). Of poisons and parasites — the defensive role of tetrodotoxin against infections in newts. Journal of Animal Ecology 87(4), 1192–1204. 29476541 10.1111/1365-2656.12816

[brv70060-bib-0108] Joshi, R. (2009). Asian elephants, *Elephas maximus* behaviour in the Rajaji National Park, North‐West India: eight years with Asian elephants. Nature and Science 7(1), 49–77.

[brv70060-bib-0109] Koch, H. , Woodward, J. , Langat, M. K. , Brown, M. J. & Stevenson, P. C. (2019). Flagellum removal by a nectar metabolite inhibits infectivity of a bumblebee parasite. Current Biology 29(20), 3494–3500. 31607528 10.1016/j.cub.2019.08.037

[brv70060-bib-0110] Korzeniowska, K. , Górka, B. , Lipok, J. & Wieczorek, P. P. (2018). Algae and their extracts in medical treatment. In Algae Biomass: Characteristics and Applications: Towards Algae‐Based Products (eds K. Chojnacka , P. P. Wieczorek , G. Schroeder and I. Michalak ), pp. 73–87. Springer, Cham.

[brv70060-bib-0111] Koyama, N. F. , Caws, C. & Aureli, F. (2006). Interchange of grooming and agonistic support in chimpanzees. International Journal of Primatology 27, 1293–1309.

[brv70060-bib-0112] Krishnamani, R. & Mahaney, W. C. (2000). Geophagy among primates: adaptive significance and ecological consequences. Animal Behaviour 59(5), 899–915. 10860518 10.1006/anbe.1999.1376

[brv70060-bib-0113] Kuswadi, A. N. (1992). Allogrooming Behavior of European Honey Bees *Apis mellifera* L. MSc Thesis, Oregon State University, USA.

[brv70060-bib-0114] Landau, S. Y. , Azaizeh, H. , Muklada, H. , Glasser, T. , Ungar, E. D. , Baram, H. , Abbas, N. & Markovics, A. (2010). Anthelmintic activity of *Pistacia lentiscus* foliage in two Middle Eastern breeds of goats differing in their propensity to consume tannin‐rich browse. Veterinary Parasitology 173(3–4), 280–286. 20705396 10.1016/j.vetpar.2010.07.006

[brv70060-bib-0115] Laumer, I. B. , Rahman, A. , Rahmaeti, T. , Azhari, U. , Hermansyah , Atmoko, S. S. U. & Schuppli, C. (2024). Active self‐treatment of a facial wound with a biologically active plant by a male Sumatran orangutan. Scientific Reports 14(1), 8932. 38698007 10.1038/s41598-024-58988-7 PMC11066025

[brv70060-bib-0116] Lazaro‐Perea, C. , Arruda, M. F. & Snowdon, C. T. (2004). Grooming as a reward? Social function of grooming between females in cooperatively breeding marmosets. Animal Behaviour 67(4), 627–636. 17237884 10.1016/j.anbehav.2003.06.004 PMC1761567

[brv70060-bib-0117] Leca, J. B. , Gunst, N. & Petit, O. (2007). Social aspects of fur‐rubbing in *Cebus capucinus* and *C. apella* . International Journal of Primatology 28(4), 801–817.

[brv70060-bib-0118] Lefèvre, T. , Chiang, A. , Kelavkar, M. , Li, H. , Li, J. , de Castillejo, C. L. F. , Oliver, L. , Potini, Y. , Hunter, M. D. & de Roode, J. C. (2012). Behavioural resistance against a protozoan parasite in the monarch butterfly. Journal of Animal Ecology 81(1), 70–79. 21939438 10.1111/j.1365-2656.2011.01901.x

[brv70060-bib-0119] Lefèvre, T. , Oliver, L. , Hunter, M. D. & de Roode, J. C. (2010). Evidence for trans‐generational medication in nature. Ecology Letters 13(12), 1485–1493. 21040353 10.1111/j.1461-0248.2010.01537.x

[brv70060-bib-0120] Leung, B. , Forbes, M. R. & Baker, R. L. (2001). Nutritional stress and behavioural immunity of damselflies. Animal Behaviour 61(6), 1093–1099.

[brv70060-bib-0121] Longino, J. T. (1984). True anting by the capuchin, *Cebus capucinus* . Primates 25, 243–245.

[brv70060-bib-0122] Lozano, G. A. (1998). Parasitic stress and self‐medication in wild animals. Advances in the Study of Behaviour 27, 291–318.

[brv70060-bib-0123] Lucon‐Xiccato, T. , Carere, C. & Baracchi, D. (2024). Intraspecific variation in invertebrate cognition: a review. Behavioral Ecology and Sociobiology 78(1), 1–18.

[brv70060-bib-0124] Lynch Alfaro, J. W. , Matthews, L. , Boyette, A. H. , Macfarlan, S. J. , Phillips, K. A. , Falótico, T. , Ottoni, E. , Verderane, M. , Izar, P. , Schulte, M. , Melin, A. , Fedigan, L. , Jason, C. & Alfaro, M. E. (2012). Anointing variation across wild capuchin populations: a review of material preferences, bout frequency and anointing sociality in *Cebus* and *Sapajus* . American Journal of Primatology 74(4), 299–314. 21769906 10.1002/ajp.20971

[brv70060-bib-0125] Macfoy, C. , Danosus, D. , Sandit, R. , Jones, T. H. , Garraffo, H. M. , Spande, T. F. & Daly, J. W. (2005). Alkaloids of anuran skin: antimicrobial function? Zeitschrift für Naturforschung. Section C 60(11–12), 932–937. 10.1515/znc-2005-11-1218 16402556

[brv70060-bib-0126] MacIntosh, A. J. J. & Huffman, M. A. (2010). Towards understanding the role of diet in host‐parasite interactions in the case of Japanese macaques. In The Japanese Macaques (eds F. Nakagawa , M. Nakamichi and H. Sugiura ), pp. 323–344. Springer, Tokyo.

[brv70060-bib-0127] Mahaney, W. C. , Hancock, R. G. V. & Aufreiter, S. (1995). Mountain gorilla geophagy: a possible strategy for dealing with intestinal problems. International Journal of Primatology 16(3), 475–487.

[brv70060-bib-0128] Mahaney, W. C. , Watts, D. P. & Hancock, R. G. V. (1990). Geophagia by mountain gorillas (*Gorilla gorilla beringei*) in the Virunga Mountains, Rwanda. Primates 31, 113–120.

[brv70060-bib-0129] Makuya, L. & Schradin, C. (2024). Costs and benefits of solitary living in mammals. Journal of Zoology 323(1), 918.

[brv70060-bib-0130] Mamillapalli, V. , Jujjavarapu, B. & Kantamneni, P. (2016). Zoo pharmacognosy: animal self‐medication. Journal of Critical Reviews 3(3), 13–17.

[brv70060-bib-0131] Manson, J. S. , Otterstatter, M. C. & Thomson, J. D. (2010). Consumption of a nectar alkaloid reduces pathogen load in bumble bees. Oecologia 162, 81–89. 19711104 10.1007/s00442-009-1431-9

[brv70060-bib-0132] Margalida, A. , Almirall, I. & Negro, J. J. (2023). New insights into the cosmetic behaviour of bearded vultures: ferruginous springs are shared sequentially. Animals 13(15), 2409. 37570218 10.3390/ani13152409 PMC10416836

[brv70060-bib-0133] Mascaro, A. , Southern, L. M. , Deschner, T. & Pika, S. (2022). Application of insects to wounds of self and others by chimpanzees in the wild. Current Biology 32(3), R112–R113. 35134354 10.1016/j.cub.2021.12.045

[brv70060-bib-0134] McCracken, S. F. & Forstner, M. R. J. (2006). Geophagy: *Bufo Margaritifer* (Anura: Bufonidae). Herpetological Review 37(2), 7273.

[brv70060-bib-0135] McLennan, M. R. & Huffman, M. A. (2012). High frequency of leaf swallowing and its relationship to parasite expulsion in “village” chimpanzees at Bulindi, Uganda. American Journal of Primatology 74(7), 642–650. 22644578 10.1002/ajp.22017

[brv70060-bib-0136] McMillan, B. R. , Cottam, M. R. & Kaufman, D. W. (2000). Wallowing behavior of American bison (*Bos bison*) in tallgrass prairie: an examination of alternate explanations. American Midland Naturalist 144(1), 159–167.

[brv70060-bib-0137] Mebs, D. , Pogoda, W. & Toennes, S. W. (2018). Loss of skin alkaloids in poison toads, *Melanophryniscus klappenbachi* (Anura: Bufonidae) when fed alkaloid‐free diet. Toxicon 150, 267–269. 29913195 10.1016/j.toxicon.2018.06.075

[brv70060-bib-0138] Medeiros, K. , Campêlo, A. , Maia, A. C. D. , Freire Filho, R. , Do Amaral Ferraz Navarro, D. M. , Chagas, A. Jr. , Bastos, M. & Bezerra, B. (2020). Wild blonde capuchins (*Sapajus flavius*) perform anointing behaviour using toxic secretions of a millipede (Spirobolida: Rhinocricidae). Journal of Chemical Ecology 46, 1010–1015. 32984924 10.1007/s10886-020-01215-0

[brv70060-bib-0139] Merchant, M. , Williams, S. , Trosclair, P. L. III , Elsey, R. M. & Mills, K. (2007). Febrile response to infection in the American alligator (*Alligator mississippiensis*). Comparative Biochemistry and Physiology Part A: Molecular & Integrative Physiology 148(4), 921–925. 10.1016/j.cbpa.2007.09.016 17977038

[brv70060-bib-0140] Mina, A. E. , Ponti, A. K. , Woodcraft, N. L. , Johnson, E. E. & Saporito, R. A. (2015). Variation in alkaloid‐ based microbial defenses of the dendrobatid poison frog *Oophaga pumilio* . Chemoecology 25, 169–178.

[brv70060-bib-0141] Moore, J. & Freehling, M. (2002). Cockroach hosts in thermal gradients suppress parasite development. Oecologia 133, 261–266. 28547314 10.1007/s00442-002-1030-5

[brv70060-bib-0142] Morozov, N. S. (2015). Why do birds practice anting? Biology Bulletin Reviews 5, 353–365.

[brv70060-bib-0143] Morrogh‐Bernard, H. C. (2008). Fur‐rubbing as a form of self‐medication in *Pongo pygmaeus* . International Journal of Primatology 29(4), 1059–1064.

[brv70060-bib-0144] Morrogh‐Bernard, H. C. , Foitová, I. , Yeen, Z. , Wilkin, P. , De Martin, R. , Rárová, L. , Doležal, K. , Nurcahyo, W. & Olšanský, M. (2017). Self‐medication by orang‐utans (*Pongo pygmaeus*) using bioactive properties of *Dracaena cantleyi* . Scientific Reports 7(1), 16653. 29192145 10.1038/s41598-017-16621-w PMC5709421

[brv70060-bib-0145] Müller, C. B. & Schmid‐Hempel, P. (1993). Exploitation of cold temperature as defence against parasitoids in bumblebees. Nature 363(6424), 65–67.

[brv70060-bib-0146] Ode, P. J. (2006). Plant chemistry and natural enemy fitness: effects on herbivore and natural enemy interactions. Annual Review of Entomology 51(1), 163–185. 10.1146/annurev.ento.51.110104.151110 16332208

[brv70060-bib-0147] Ontiveros, D. , Caro, J. & Pleguezuelos, J. M. (2008). Green plant material versus ectoparasites in nests of Bonelli's eagle. Journal of Zoology 274(1), 99–104.

[brv70060-bib-0148] Opitz, S. E. & Müller, C. (2009). Plant chemistry and insect sequestration. Chemoecology 19, 117–154.

[brv70060-bib-0149] Otti, O. , Tragust, S. & Feldhaar, H. (2014). Unifying external and internal immune defences. Trends in Ecology & Evolution 29(11), 625–634. 25278329 10.1016/j.tree.2014.09.002

[brv70060-bib-0150] Ouedraogo, R. M. , Cusson, M. , Goettel, M. S. & Brodeur, J. (2003). Inhibition of fungal growth in thermoregulating locusts, *Locusta migratoria*, infected by the fungus *Metarhizium anisopliae* var *acridum* . Journal of Invertebrate Pathology 82(2), 103–109. 12623310 10.1016/s0022-2011(02)00185-4

[brv70060-bib-0151] Ouedraogo, R. M. , Goettel, M. S. & Brodeur, J. (2004). Behavioral thermoregulation in the migratory locust: a therapy to overcome fungal infection. Oecologia 138, 312–319. 14614620 10.1007/s00442-003-1431-0

[brv70060-bib-0152] Page, J. E. , Huffman, M. A. , Smith, V. & Towers, G. H. N. (1997). Chemical basis for *Aspilia* leaf‐swallowing by chimpanzees: a re‐analysis. Journal of Chemical Ecology 23(9), 2211–2225.

[brv70060-bib-0153] Panagiotakopulu, E. , Buckland, P. C. , Day, P. M. , Sarpaki, A. & Doumas, C. (1995). Natural insecticides and insect repellents in antiquity: a review of the evidence. Journal of Archaeological Science 22(5), 705–710.

[brv70060-bib-0154] Pandey, H. P. & Verma, A. K. (2017). A study on the role of holy basil (*Ocimum sanctum*) in auto‐healing of Indian garden lizard (*Calotes versicolor*). International Journal of Fauna and Biological Studies 4(2), 97–100.

[brv70060-bib-0155] Papastamatiou, Y. P. , Meyer, C. G. & Maragos, J. E. (2007). Sharks as cleaners for reef fish. Coral Reefs 26, 277.

[brv70060-bib-0156] Pasmans, F. , Van Rooij, P. , Blooi, M. , Tessa, G. , Bogaerts, S. , Sotgiu, G. , Garner, T. W. J. , Fisher, M. C. , Schmidt, B. R. , Woeltjes, T. , Beukema, W. , Bovero, S. , Adriaensen, C. , Oneto, F. , Ottonello, D. , *et al*. (2013). Resistance to chytridiomycosis in European plethodontid salamanders of the genus *Speleomantes* . PLoS One 8(5), e63639. 23703511 10.1371/journal.pone.0063639 PMC3659026

[brv70060-bib-0157] Pawlik, J. R. , Kernan, M. R. , Molinski, T. F. , Harper, M. K. & Faulkner, D. J. (1988). Defensive chemicals of the Spanisch dancer nudibranch *Hexabranchus sanguineus* and its egg ribbons: macrolides derived from a sponge diet. Journal of Experimental Marine Biology and Ecology 119(2), 99–109.

[brv70060-bib-0158] Pebsworth, P. A. , Huffman, M. A. , Lambert, J. E. & Young, S. L. (2019). Geophagy among nonhuman primates: a systematic review of current knowledge and suggestions for future directions. American Journal of Physical Anthropology 168, 164–194. 30508222 10.1002/ajpa.23724

[brv70060-bib-0159] Peckre, L. R. , Defolie, C. , Kappeler, P. M. & Fichtel, C. (2018). Potential self‐medication using millipede secretions in red‐fronted lemurs: combining anointment and ingestion for a joint action against gastrointestinal parasites? Primates 59, 483–494. 30058024 10.1007/s10329-018-0674-7

[brv70060-bib-0160] Phillips‐Conroy, J. E. (1986). Baboons, diet and disease: food selection and schistosomiasis. In Current Perspectives in Primate Social Dynamics (eds D. M. Taub and F. A. King ), pp. 287–304. Van Nostrand Reinhold, New York, NY.

[brv70060-bib-0161] Pull, C. D. , Metzler, S. , Naderlinger, E. & Cremer, S. (2018). Protection against the lethal side effects of social immunity in ants. Current Biology 28(19), R1139–R1140. 30300596 10.1016/j.cub.2018.08.063

[brv70060-bib-0162] Pusceddu, M. , Annoscia, D. , Floris, I. , Frizzera, D. , Zanni, V. , Angioni, A. , Satta, A. & Nazzi, F. (2021 *a*). Honeybees use propolis as a natural pesticide against their major ectoparasite. Proceedings of the Royal Society B 288(1965), 20212101. 34905714 10.1098/rspb.2021.2101 PMC8670950

[brv70060-bib-0163] Pusceddu, M. , Cini, A. , Alberti , S. , Salaris, E. , Theodorou, P. , Floris, I. & Satta, A. (2021 *b*). Honey bees increase social distancing when facing the ectoparasite *Varroa destructor* . Science Advances 7(44), eabj1398. 34714677 10.1126/sciadv.abj1398 PMC8555907

[brv70060-bib-0164] Pusceddu, M. , Floris, I. , Mura, A. , Theodorou, P. , Cirotto, G. , Piluzza, G. , Bullitta, S. , Angioni, A. & Satta, A. (2018). The effects of raw propolis on *Varroa*‐infested honey bee (*Apis mellifera*) workers. Parasitology Research 117, 3527–3535. 30120588 10.1007/s00436-018-6050-0

[brv70060-bib-0165] Pusceddu, M. , Piluzza, G. , Theodorou, P. , Buffa, F. , Ruiu, L. , Bullitta, S. , Floris, I. & Satta, A. (2019). Resin foraging dynamics in *Varroa destructor*‐infested hives: a case of medication of kin? Insect Science 26(2), 297–310. 28795524 10.1111/1744-7917.12515

[brv70060-bib-0166] Quicke, D. L. J. , Ghafouri Moghaddam, M. & Butcher, B. A. (2023). Dietary challenges for parasitoid (Hymenoptera: Ichneumonoidea); coping with toxic hosts, or not? Toxins 15(7), 424. 37505693 10.3390/toxins15070424 PMC10467097

[brv70060-bib-0167] Rajasekar, R. , Chattopadhyay, B. & Sripathi, K. (2006). Depositing masticated plant materials inside tent roosts in *Cynopterus sphinx* (Chiroptera: Pteropodidae) in Southern India. Acta Chiropterologica 8(1), 269–274.

[brv70060-bib-0168] Rakus, K. , Ronsmans, M. & Vanderplasschen, A. (2017). Behavioral fever in ectothermic vertebrates. Developmental & Comparative Immunology 66, 84–91. 27381718 10.1016/j.dci.2016.06.027

[brv70060-bib-0169] Rees, P. A. (2002). Asian elephants (*Elephas maximus*) dust bathe in response to an increase in environmental temperature. Journal of Thermal Biology 27(5), 353–358.

[brv70060-bib-0170] Reynolds, S. J. , Ibáñez‐Álamo, J. D. , Sumasgutner, P. & Mainwaring, M. C. (2019). Urbanisation and nest building in birds: a review of threats and opportunities. Journal of Ornithology 160(3), 841–860.

[brv70060-bib-0171] Richardson, L. L. , Adler, L. S. , Leonard, A. S. , Andicoechea, J. , Regan, K. H. , Anthony, W. E. , Manson, J. S. & Irwin, R. E. (2015). Secondary metabolites in floral nectar reduce parasite infections in bumblebees. Proceedings of the Royal Society B: Biological Sciences 282(1803), 20142471. 10.1098/rspb.2014.2471 PMC4345440 25694627

[brv70060-bib-0172] Ritter, E. K. (2011). Use of sand ripples to enhance chafing in Caribbean reef sharks (*Carcharhinus perezi*) and blacktip sharks (*Carcharhinus limbatus*). Bulletin of Marine Science 87(3), 413–419.

[brv70060-bib-0173] Rodgman, A. & Perfetti, T. A. (2013). The Chemical Components of Tobacco and Tobacco Smoke, 2nd Edition. CRC Press, Boca Raton, FL.

[brv70060-bib-0174] Rodríguez, C. , Rollins‐Smith, L. , Ibáñez, R. , Durant‐Archibold, A. A. & Gutiérrez, M. (2017). Toxins and pharmacologically active compounds from species of the family Bufonidae (Amphibia, Anura). Journal of Ethnopharmacology 198, 235–254. 28034659 10.1016/j.jep.2016.12.021

[brv70060-bib-0175] Rodriguez, E. , Aregullin, M. , Nishida, T. , Uehara, S. , Wrangham, R. , Abramowski, Z. , Finlayson, A. & Towers, G. H. N. (1985). Thiarubrine A, a bioactive constituent of *Aspilia* (Asteraceae) consumed by wild chimpanzees. Experientia 41, 419–420. 3972092 10.1007/BF02004537

[brv70060-bib-0176] Rodriguez, E. & Wrangham, R. (1993). Zoopharmacognosy: the use of medicinal plants by animals. In Phytochemical Potential of Tropical Plants (eds K. R. Downum , J. T. Romeo and H. A. Stafford ), pp. 89–105. Springer, Boston, MA.

[brv70060-bib-0177] Rollins‐Smith, L. A. (2009). The role of amphibian antimicrobial peptides in protection of amphibians from pathogens linked to global amphibian declines. Biochimica et Biophysica Acta (BBA) ‐ Biomembranes 1788(8), 1593–1599. 19327341 10.1016/j.bbamem.2009.03.008

[brv70060-bib-0178] Sachs, B. D. (1988). The development of grooming and its expression in adult animals. Annals of the New York Academy of Sciences 525(1), 1–17. 10.1111/j.1749-6632.1988.tb38591.x 3291663

[brv70060-bib-0179] Salmela, H. , Amdam, G. V. & Freitak, D. (2015). Transfer of immunity from mother to offspring is mediated via egg‐yolk protein vitellogenin. PLoS Pathogens 11(7), e1005015. 26230630 10.1371/journal.ppat.1005015 PMC4521805

[brv70060-bib-0180] Sánchez, K. F. , Huntley, N. , Duffy, M. A. & Hunter, M. D. (2019). Toxins or medicines? Phytoplankton diets mediate host and parasite fitness in a freshwater system. Proceedings of the Royal Society B 286(1894), 20182231. 30963882 10.1098/rspb.2018.2231 PMC6367176

[brv70060-bib-0181] Sapolsky, R. M. (1994). Fallible instinct: a dose of skepticism about the medicinal “knowledge” of animals. Sciences 34(1), 13–15.

[brv70060-bib-0182] Saporito, R. A. , Donnelly, M. A. , Spande, T. F. & Garraffo, H. M. (2012). A review of chemical ecology in poison frogs. Chemoecology 22, 159–168.

[brv70060-bib-0183] Saporito, R. A. , Russell, M. W. , Richards‐Zawacki, C. L. & Dugas, M. B. (2019). Experimental evidence for maternal provisioning of alkaloid defenses in a dendrobatid frog. Toxicon 161, 40–43. 30790578 10.1016/j.toxicon.2019.02.008

[brv70060-bib-0184] Saporito, R. A. , Spande, T. F. , Garraffo, H. M. & Donnelly, M. A. (2009). Arthropod alkaloids in poison frogs: a review of the “dietary hypothesis”. Heterocycles 79(1), 277–297.

[brv70060-bib-0185] Saporito, R. A. , Zuercher, R. , Roberts, M. , Gerow, K. G. & Donnelly, M. A. (2007). Experimental evidence for aposematism in the dendrobatid poison frog *Oophaga pumilio* . Copeia 2007(4), 1006–1011.

[brv70060-bib-0186] Sauer, E. L. , Trejo, N. , Hoverman, J. T. & Rohr, J. R. (2019). Behavioural fever reduces ranaviral infection in toads. Functional Ecology 33(11), 2172–2179. 33041425 10.1111/1365-2435.13427 PMC7546308

[brv70060-bib-0187] Savitzky, A. H. , Mori, A. , Hutchinson, D. A. , Saporito, R. A. , Burghardt, G. M. , Lillywhite, H. B. & Meinwald, J. (2012). Sequestered defensive toxins in tetrapod vertebrates: principles, patterns, and prospects for future studies. Chemoecology 22, 141–158. 22904605 10.1007/s00049-012-0112-z PMC3418492

[brv70060-bib-0188] Schino, G. (2001). Grooming, competition and social rank among female primates: a meta‐analysis. Animal Behaviour 62(2), 265–271.

[brv70060-bib-0189] Schino, G. (2007). Grooming and agonistic support: a meta‐analysis of primate reciprocal altruism. Behavioral Ecology 18(1), 115–120.

[brv70060-bib-0190] Scott‐Baumann, J. F. & Morgan, E. R. (2015). A review of the nest protection hypothesis: does inclusion of fresh green plant material in birds' nests reduce parasite infestation? Parasitology 142(8), 1016–1023. 25804728 10.1017/S0031182015000189

[brv70060-bib-0191] Shurkin, J. (2014). Animals that self‐medicate. Proceedings of the National Academy of Sciences 111(49), 17339–17341. 10.1073/pnas.1419966111 PMC4267359 25492915

[brv70060-bib-0192] Simmons, K. E. L. (1957). A review of the anting‐behaviour of passerine birds. British Birds 50(10), 401–424.

[brv70060-bib-0193] Simone, M. , Evans, J. D. & Spivak, M. (2009). Resin collection and social immunity in honey bees. Evolution 63(11), 3016–3022. 19619221 10.1111/j.1558-5646.2009.00772.x

[brv70060-bib-0194] Simone‐Finstrom, M. , Borba, R. S. , Wilson, M. & Spivak, M. (2017). Propolis counteracts some threats to honey bee health. Insects 8(2), 46. 28468244 10.3390/insects8020046 PMC5492060

[brv70060-bib-0195] Simone‐Finstrom, M. & Spivak, M. (2010). Propolis and bee health: the natural history and significance of resin use by honey bees. Apidologie 41(3), 295–311.

[brv70060-bib-0196] Simone‐Finstrom, M. & Spivak, M. (2012). Increased resin collection after parasite challenge: a case of self‐medication in honey bees? PLoS One 7(3), e34601. 22479650 10.1371/journal.pone.0034601 PMC3315539

[brv70060-bib-0197] Singer, M. S. , Mace, K. C. & Bernays, E. A. (2009). Self‐medication as adaptive plasticity: increased ingestion of plant toxins by parasitized caterpillars. PLoS One 4(3), e4796. 19274098 10.1371/journal.pone.0004796 PMC2652102

[brv70060-bib-0198] Smith, H. K. , Pasmans, F. , Dhaenens, M. , Deforce, D. , Bonte, D. , Verheyen, K. , Lens, L. & Martel, A. (2018). Skin mucosome activity as an indicator of *Batrachochytrium salamandrivorans* susceptibility in salamanders. PLoS One 13(7), e0199295. 30020936 10.1371/journal.pone.0199295 PMC6051575

[brv70060-bib-0199] Sokol, O. M. (1971). Lithophagy and geophagy in reptiles. Journal of Herpetology 5(1/2), 69–71.

[brv70060-bib-0200] Spivak, M. , Goblirsch, M. & Simone‐Finstrom, M. (2019). Social‐medication in bees: the line between individual and social regulation. Current Opinion in Insect Science 33, 49–55. 31358195 10.1016/j.cois.2019.02.009

[brv70060-bib-0201] Spruijt, B. M. , Van Hooff, J. A. & Gispen, W. H. (1992). Ethology and neurobiology of grooming behavior. Physiological Reviews 72(3), 825–852. 1320764 10.1152/physrev.1992.72.3.825

[brv70060-bib-0202] Starks, P. T. , Blackie, C. A. & Seeley, T. D. (2000). Fever in honeybee colonies. Naturwissenschaften 87, 229–231. 10883439 10.1007/s001140050709

[brv70060-bib-0203] Stockmaier, S. , Stroeymeyt, N. , Shattuck, E. C. , Hawley, D. M. , Meyers, L. A. & Bolnick, D. I. (2021). Infectious diseases and social distancing in nature. Science 371(6533), abc8881. 10.1126/science.abc8881 33674468

[brv70060-bib-0204] Stroeymeyt, N. , Grasse, A. V. , Crespi, A. , Mersch, D. P. , Cremer, S. & Keller, L. (2018). Social network plasticity decreases disease transmission in a eusocial insect. Science 362(6417), 941–945. 30467168 10.1126/science.aat4793

[brv70060-bib-0205] Stynoski, J. L. , Shelton, G. & Stynoski, P. (2014 *a*). Maternally derived chemical defences are an effective deterrent against some predators of poison frog tadpoles (*Oophaga pumilio*). Biology Letters 10(5), 20140187. 24850895 10.1098/rsbl.2014.0187 PMC4046375

[brv70060-bib-0206] Stynoski, J. L. , Torres‐Mendoza, Y. , Sasa‐Marin, M. & Saporito, R. A. (2014 *b*). Evidence of maternal provisioning of alkaloid‐based chemical defenses in the strawberry poison frog *Oophaga pumilio* . Ecology 95(3), 587–593. 24804437 10.1890/13-0927.1

[brv70060-bib-0207] Su, H. , Su, Y. & Huffman, M. A. (2013). Leaf‐swallowing and parasite infection in the Chinese lesser civet (*Viverricula indica*) in northern Taiwan. Zoological Studies 52(3), 22.

[brv70060-bib-0208] Suárez‐Rodríguez, M. & Garcia, C. M. (2014). There is no such thing as a free cigarette; lining nests with discarded butts brings short‐term benefits, but causes toxic damage. Journal of Evolutionary Biology 27(12), 2719–2726. 25403778 10.1111/jeb.12531

[brv70060-bib-0209] Suárez‐Rodríguez, M. & Garcia, C. M. (2017). An experimental demonstration that house finches add cigarette butts in response to ectoparasites. Journal of Avian Biology 48(10), 1316–1321.

[brv70060-bib-0210] Suárez‐Rodríguez, M. , López‐Rull, I. & Garcia, C. M. (2013). Incorporation of cigarette butts into nests reduces nest ectoparasite load in urban birds: new ingredients for an old recipe? Biology Letters 9(1), 20120931. 23221874 10.1098/rsbl.2012.0931 PMC3565511

[brv70060-bib-0211] Tanaka, I. (1995). Matrilineal distribution of louse egg‐handling techniques during grooming in free‐ranging Japanese macaques. American Journal of Physical Anthropology 98(2), 197–201. 8644879 10.1002/ajpa.1330980208

[brv70060-bib-0212] Tao, L. , Hoang, K. M. , Hunter, M. D. & de Roode, J. C. (2016). Fitness costs of animal medication: antiparasitic plant chemicals reduce fitness of monarch butterfly hosts. Journal of Animal Ecology 85(5), 1246–1254. 27286503 10.1111/1365-2656.12558

[brv70060-bib-0213] Tasdemir, D. , MacIntosh, A. J. J. , Stergioua, P. , Kaisere, M. , Mansourg, N. R. , Bickleg, Q. & Huffman, M. A. (2020). Antiprotozoal and antihelminthic properties of plants ingested by wild Japanese macaques (*Macaca fuscata yakui*) in Yakushima Island. Journal of Ethnopharmacology 247, 112270. 31589965 10.1016/j.jep.2019.112270

[brv70060-bib-0214] Terebiznik, M. , Moldowan, P. D. , Leivesley, J. A. , Massey, M. D. , Lacroix, C. , Connoy, J. W. & Rollinson, N. (2020). Hatchling turtles ingest natural and artificial incubation substrates at high frequency. Behavioral Ecology and Sociobiology 74, 1–12.

[brv70060-bib-0215] Thompson, C. D. & Meeuwig, J. J. (2022). Sharks are the preferred scraping surface for large pelagic fishes: possible implications for parasite removal and fitness in a changing ocean. PLoS One 17(10), e0275458. 36260545 10.1371/journal.pone.0275458 PMC9581428

[brv70060-bib-0216] Tiddi, B. , Aureli, F. & Schino, G. (2012). Grooming up the hierarchy: the exchange of grooming and rank‐related benefits in a new world primate. PLoS One 7(5), e36641. 22590582 10.1371/journal.pone.0036641 PMC3348124

[brv70060-bib-0217] Torres‐Fajardo, R. A. , González‐Pech, P. G. , Sandoval‐Castro, C. A. & Torres‐Acosta, J. F. K. (2019). Criollo goats limit their grass intake in the early morning suggesting a prophylactic self‐medication behaviour in a heterogeneous vegetation. Tropical Animal Health and Production 51, 2473–2479. 31197723 10.1007/s11250-019-01966-3

[brv70060-bib-0218] Tragust, S. , Mitteregger, B. , Barone, V. , Konrad, M. , Ugelvig, L. V. & Cremer, S. (2013). Ants disinfect fungus‐exposed brood by oral uptake and spread of their poison. Current Biology 23(1), 76–82. 23246409 10.1016/j.cub.2012.11.034

[brv70060-bib-0219] Tributsch, H. (2016). Ochre bathing of the bearded vulture: a bio‐mimetic model for early humans towards smell prevention and health. Animals 6(1), 7. 26784238 10.3390/ani6010007 PMC4730124

[brv70060-bib-0220] Uenoyama, R. , Miyazaki, T. , Hurst, J. L. , Beynon, R. J. , Adachi, M. , Murooka, T. , Onoda, I. , Miyazawa, Y. , Katayama, R. , Yamashita, T. , Kaneko, S. , Nishikawa, T. & Miyazaki, M. (2021). The characteristic response of domestic cats to plant iridoids allows them to gain chemical defense against mosquitoes. Science Advances 7(4), eabd9135. 33523929 10.1126/sciadv.abd9135 PMC7817105

[brv70060-bib-0221] Vaelli, P. M. , Theis, K. R. , Williams, J. E. , O'Connell, L. A. , Foster, J. A. & Eisthen, H. L. (2020). The skin microbiome facilitates adaptive tetrodotoxin production in poisonous newts. eLife 9, e53898. 32254021 10.7554/eLife.53898 PMC7138609

[brv70060-bib-0222] Valderrama, X. , Robinson, J. G. , Attygalle, A. B. & Eisner, T. (2000). Seasonal anointment with millipedes in a wild primate: a chemical defense against insects? Journal of Chemical Ecology 26, 2781–2790.

[brv70060-bib-0223] Vander Meer, R. K. & Morel, L. (1995). Ant queens deposit pheromones and antimicrobial agents on eggs. Naturwissenschaften 82, 93–95.

[brv70060-bib-0224] Verderane, M. P. , Falótico, T. , Resende, B. D. , Labruna, M. B. , Izar, P. & Ottoni, E. B. (2007). Anting in a semifree‐ranging group of *Cebus apella* . International Journal of Primatology 28, 47–53.

[brv70060-bib-0225] Villalba, J. J. , Miller, J. , Ungar, E. D. , Landau, S. Y. & Glendinning, J. (2014). Ruminant self‐medication against gastrointestinal nematodes: evidence, mechanism, and origins. Parasite 21, 31. 24971486 10.1051/parasite/2014032 PMC4073621

[brv70060-bib-0226] Villalba, J. J. & Provenza, F. D. (2007). Self‐medication and homeostatic behaviour in herbivores: learning about the benefits of nature's pharmacy. Animal 1(9), 1360–1370. 22444892 10.1017/S1751731107000134

[brv70060-bib-0227] Villalba, J. J. , Provenza, F. D. & Shaw, R. (2006). Sheep self‐medicate when challenged with illness‐inducing foods. Animal Behaviour 71(5), 1131–1139.

[brv70060-bib-0228] Villanueva, E. D. , Brooks, O. L. , Bolton, S. K. , Savastano, N. , Schulte, L. M. & Saporito, R. A. (2022). Maternal provisioning of alkaloid defenses are present in obligate but not facultative egg feeding dendrobatids. Journal of Chemical Ecology 48(11–12), 900–909. 36564635 10.1007/s10886-022-01394-y

[brv70060-bib-0229] Viviano, A. , Huffman, M. A. , Senini, C. & Mori, E. (2022). Do porcupines self‐medicate? The seasonal consumption of plants with antiparasitic properties coincides with that of parasite infections in *Hystrix cristata* of Central Italy. European Journal of Wildlife Research 68(6), 72.

[brv70060-bib-0230] Weldon, P. J. , Aldrich, J. R. , Klun, J. A. , Oliver, J. E. & Debboun, M. (2003). Benzoquinones from millipedes deter mosquitoes and elicit self‐anointing in capuchin monkeys (*Cebus spp*.). Naturwissenschaften 90, 301–304. 12883771 10.1007/s00114-003-0427-2

[brv70060-bib-0231] Weldon, P. J. & Carroll, J. F. (2006). Vertebrate chemical defense: secreted and topically acquired deterrents of arthropods. In Insect Repellents: Principles, Methods, and Users (eds M. Debboun , S. P. Frances and D. Strickman ), pp. 47–74. CRC Press, Boca Raton, FL.

[brv70060-bib-0232] Weldon, P. J. , Kramer, M. , Gordon, S. , Spande, T. F. & Daly, J. W. (2006). A common pumiliotoxin from poison frogs exhibits enantioselective toxicity against mosquitoes. Proceedings of the National Academy of Sciences 103(47), 17818–17821. 10.1073/pnas.0608646103 PMC1693830 17095598

[brv70060-bib-0233] Williams, B. L. , Hanifin, C. T. , Brodie, E. D. & Caldwell, R. L. (2011). Ontogeny of tetrodotoxin levels in blue‐ringed octopuses: maternal investment and apparent independent production in offspring of *Hapalochlaena lunulata* . Journal of Chemical Ecology 37, 10–17. 21165679 10.1007/s10886-010-9901-4

[brv70060-bib-0234] Williams, L. H. , Anstett, A. , Bach Muñoz, V. , Chisholm, J. , Fallows, C. , Green, J. R. , Higuera rivas, J. E. , Skomal, G. , Winton, M. & Hammerschlag, N. (2022). Sharks as exfoliators: widespread chafing between marine organisms suggests an unexplored ecological role. Ecology 103(1), e03570. 34709650 10.1002/ecy.3570

[brv70060-bib-0235] Wilson, S. N. , Sindi, S. S. , Brooks, H. Z. , Hohn, M. E. , Price, C. R. , Radunskaya, A. E. , Williams, N. D. & Fefferman, N. H. (2020). How emergent social patterns in allogrooming combat parasitic infections. Frontiers in Ecology and Evolution 8, 54.

[brv70060-bib-0236] Wilson‐Rich, N. , Spivak, M. , Fefferman, N. H. & Starks, P. T. (2009). Genetic, individual, and group facilitation of disease resistance in insect societies. Annual Review of Entomology 54(1), 405–423. 10.1146/annurev.ento.53.103106.093301 18793100

[brv70060-bib-0237] Wimberger, P. H. (1984). The use of green plant material in bird nests to avoid ectoparasites. The Auk 101(3), 615–618.

[brv70060-bib-0238] Wrangham, R. W. (1995). Relationship of chimpanzee leaf‐swallowing to a tapeworm infection. American Journal of Primatology 37(4), 297–303. 31936954 10.1002/ajp.1350370404

[brv70060-bib-0239] Wrangham, R. W. & Nishida, T. (1983). *Aspilia* spp. leaves: a puzzle in the feeding behavior of wild chimpanzees. Primates 24(2), 276–282.

[brv70060-bib-0240] Zamma, K. (2002). Grooming site preferences determined by lice infection among Japanese macaques in Arashiyama. Primates 43(1), 41–49. 12091746 10.1007/BF02629575

[brv70060-bib-0241] Zembrzuski, D. , Woller, D. A. , Jaronski, S. , Black, L. R. , Reuter, K. C. , Grief, D. , Beatty, A. & Cease, A. J. (2023). Understanding how diet and temperature affect survival and subsequent sporulation in a major rangeland grasshopper pest, *Melanoplus sanguinipes*, infected with the entomopathogenic fungus, Metarhizium robertsii. Biological Control 183, 105268.

